# Synthesis, structural characterization, and dual DNA/HSA binding of novel Palladium(II) violurate complex with selective p53/Caspase-3-mediated anticancer activity

**DOI:** 10.1038/s41598-026-58248-w

**Published:** 2026-06-27

**Authors:** Maged A. El-Kemary, Abd El-Motaleb, M. Ramadan, Heba A. Sahyon, Asmaa A. Shabbat, Ahmed M. Fathy

**Affiliations:** 1 Institute of Nanoscience and Nanotechnology, Kafr El-Sheikh University, Kafr El, Sheikh, 33511 Egypt; 2 Chemistry Department, Faculty of Science, Kafr El-Sheikh University, Kafr El, Sheikh, 33511 Egypt; 3 Chemistry Department, Faculty of Science, Zagazig University, Zagazig, Egypt

**Keywords:** Palladium(II) violurate complex, PXRD structural analysis, DNA/HSA binding, Anticancer, DFT, Biochemistry, Cancer, Chemical biology, Chemistry, Computational biology and bioinformatics, Drug discovery

## Abstract

A novel palladium(II) complex with the violurate ligand, [Pd(H₂L)₂], was synthesized and characterized. Molar conductivity (15.55 Ω^-1^ cm² mol^-1^) confirmed its non-electrolytic nature, and ESI-MS showed a molecular ion peak at m/z 417.57 matching [C₈H₄N₆O₈Pd]⁺. PXRD structural analysis revealed a triclinic crystal system (space group P-1) with τ₄ = 0.04, confirming square-planar geometry around Pd(II), with Pd–O bond lengths ranging from 2.065 to 2.178 Å. Thermal analysis showed three decomposition stages (41.00%, 15.00%, and 18.50% mass losses) with palladium metal as the final residue. The complex exhibited DNA binding constants of K_b_ = 1.694 × 10⁴ M^-1^ (UV-Vis) and 2.870 × 10^7^ M^-1^ (fluorescence) with ΔG = -24.12 and − 42.94 kJ mol^-1^, respectively, indicating groove binding mode as confirmed by viscosity measurements. HSA binding constants were K_b_ = 2.784 × 10^5^ M^-1^ (UV-Vis) and 6.546 × 10^9^ M^-1^ (fluorescence). Cytotoxicity assays against MDA-MB-231, HCT-116, and HepG-2 cell lines gave IC₅₀ values of 29.42, 38.11, and 24.68 µM, respectively, with selectivity indices (1.99, 1.78, and 1.29) higher than cisplatin. The complex induced late apoptosis (8.46-fold increase) via p53/caspase-3 upregulation (4.46-fold and 3.96-fold, respectively). DFT calculations showed reduced HOMO-LUMO gap (5.406 eV vs. 6.517 eV for free ligand), correlating with enhanced bioactivity. These findings present [Pd(H₂L)₂] complex as a promising dual-targeting anticancer candidate. Unlike conventional Pd(II) complexes that target DNA via intercalation or N/S coordination, our complex employs an unusual O₄ coordination sphere from violurate ligands, demonstrating a non-intercalative groove-binding mode and a p53/caspase-3 mediated apoptosis with enhanced selectivity index.

**Supplementary Information:**

The online version contains supplementary material available at 10.1038/s41598-026-58248-w.

## Introduction

The design of metal complexes as anticancer agents remains a central focus in bioinorganic and medicinal chemistry^1^. The biological activity of such complexes often stems from their capacity to bind and disrupt cellular DNA, thereby inducing cytotoxic effects^2^. Through interactions such as intercalation, groove binding, or covalent adduct formation, metallodrugs can interfere with essential DNA processes, such as replication and transcription, ultimately leading to cell cycle arrest and apoptosis in malignant cells^3,4^. Consequently, optimizing the DNA-binding affinity and selectivity of these compounds is crucial for enhancing therapeutic efficacy while reducing adverse effects on healthy tissues^5,6^.

In addition to DNA interactions, the pharmacokinetic profile and targeted delivery of metallodrugs are vital for their anticancer success. Human serum albumin (HSA), the most abundant plasma protein, serves as a principal transport vehicle in the bloodstream^7^. Strong reversible binding to HSA can enhance drug solubility, prolong circulation time, and promote accumulation in tumor tissues through mechanisms such as the enhanced permeability and retention (EPR) effect^7^. Thus, developing complexes with dual affinity for both DNA and HSA represents a promising strategy to improve drug efficacy and selectivity^8,9^.

The clinical success of cisplatin and related platinum(II)-based agents has fundamentally shaped modern metal-based chemotherapy. However, their utility is often limited by significant drawbacks, including severe dose-limiting toxicities, intrinsic or acquired drug resistance, and inactivity against certain carcinomas^10^. These limitations have motivated the ongoing exploration of alternative transition metal complexes with distinct physicochemical properties and mechanisms of action. Among these, palladium(II) complexes represent an encouraging class of experimental antineoplastic agents^11,12^. Their square-planar geometry, faster ligand exchange kinetics compared to platinum analogues, and versatile coordination chemistry enable the design of structures with tailored reactivity and diverse biological targets^13^. Notably, palladium(II) complexes can circumvent classical cisplatin resistance pathways and often exhibit potent cytotoxicity against both chemotherapy-resistant and sensitive human cancer cells^14,15^. Although further investigation into their in vivo stability and toxicity profiles is required for clinical translation, preliminary studies underscore their considerable potential as an innovative strategy to overcome the limitations of conventional platinum-based chemotherapy.

Among various ligand systems, oxime-based ligands have attracted interest due to their versatile coordination modes and notable biological activities^16^. Violuric acid (5-isonitrosobarbituric acid) is a particularly interesting oxime derivative characterized by an extended π-conjugated barbiturate core and multiple donor sites^17,18^. These structural features enhance metal-binding capability and allow for tautomerism and rich redox chemistry, which may influence the biological behavior of its metal complexes^18–20^. While some metal-violurate systems have been studied for their material and catalytic properties, their potential as anticancer agents remains largely unexplored^19,21^. In the same way, their ability to bind to DNA creates opportunities for developing new therapeutic and diagnostic agents^22–24^.

While metal-violurate systems have been explored for material and catalytic properties^19,21^, and several Pd(II) complexes have been reported with various ligands^11–15,25–32^, the combination of violuric acid with palladium(II) for dual DNA/HSA targeting with mechanistic apoptotic studies remains unexplored. To the best of our knowledge, this work presents: (i) the first comprehensive structural elucidation of a Pd(II)-violurate complex using Rietveld-refined PXRD with CCDC deposition, (ii) the first demonstration of the p53/caspase-3 apoptotic pathway for this complex type, (iii) complete thermodynamic profiles for both DNA and HSA binding, (iv) direct correlation between DFT-based reactivity descriptors and experimental bioactivity, and (v) a selectivity index analysis demonstrating improved safety compared to cisplatin. These features distinguish our study from previous reports on violurate-metal complexes^18,33,34^ and general Pd(II) anticancer agents^12,14,31^.

This study aims to synthesize and characterize a novel palladium(II) complex with violuric acid and systematically evaluate its potential as an anticancer agent. The investigation will focus on examining its DNA- and HSA-binding properties using spectroscopic and computational techniques, alongside in vitro assessments of its cytotoxicity against selected human cancer cell lines. To the best of our knowledge, this work presents a detailed investigation of a palladium(II) violurate complex, with an emphasis on its dual DNA/HSA interaction profile and its apoptosis-inducing pathway via the p53/caspase-3 cascade. While some metal-violurate systems have been explored for material and catalytic properties, their potential as anticancer agents remains relatively underexplored. The unique tautomeric and coordination features of the violurate ligand are anticipated to confer distinct reactivity and selectivity, offering new insights into the design of targeted metallodrugs.

## Experimental

### Chemicals and materials

All chemical reagents were obtained from commercial suppliers (Sigma-Aldrich, Merck, and BDH) and used without further purification. The following materials were used: potassium tetrachloropalladate(II) (K₂[PdCl₄], ≥ 99.9%), violuric acid monohydrate (C₄H₃N₃O₄·H₂O, ≥ 98%), dimethyl sulfoxide (DMSO, ≥ 99.5%), dimethyl formamide (DMF, ≥ 99.8%), ethanol absolute (≥ 99.8%), diethyl ether (≥ 99.5%), calf thymus DNA (ct-DNA, highly polymerized), human serum albumin (HSA, ≥ 96%), and all other solvents of analytical grade. Tris-HCl buffer (pH 7.4) was prepared using doubly distilled water.

### Synthesis of [Pd(H₂L)₂] complex

An aqueous solution of K₂[PdCl₄] (0.02 M, 25 mL) was added dropwise to a heated ethanolic solution (30 mL, 60 °C) of violuric acid (0.04 M, molar ratio 1:2) under continuous stirring. The mixture was refluxed for one hour at 80 °C (oil bath temperature), following a previously reported method with slight modifications^33^ yielding an orange-red precipitate. After cooling to room temperature, the product was collected by filtration, washed with hot ethanol (3 × 10 mL) and diethyl ether (2 × 10 mL), then dried over CaO for 72 h. The yield was 85.6% (0.178 g based on Pd). The synthesis was repeated five times with yields ranging from 84.2% to 86.1%, confirming excellent reproducibility. The purity of the synthesized compound was verified through elemental analysis and a measured m/z value of 417.57 (Table 1).

### Physicochemical characterization

The infrared spectra were recorded using KBr disks in the 4000–400 cm^− 1^ range on the Jusco FT/IR-4100 type A FTIR spectrometer, which recorded an infrared spectrum in transmission mode (%T) with 24 scans, a range of 199.193–4001.57 cm^‒1^, a resolution of 8 cm^‒1^, and a TGS detector. The electronic absorption spectra were obtained in DMF solution with a Shimadzu UV-2450 spectrophotometer.

The specific conductance of the complex was measured using a freshly prepared 10^− 3^ M solution in electrochemically DMF at room temperature, using a YSI Model 32 conductance meter.

The thermogravimetric measurements were performed using a Shimadzu TG 50-Thermogravimetric analyzer in the 25–1100 °C range and under an N_2_ atmosphere. Elemental analyses were carried out at the Microanalytical Unit of Cairo University. The electron-impact mass spectra were obtained on an MX-1320 instrument.

The powder X-ray diffraction spectra of the solid microcrystalline samples of the Pd(II) complex were obtained using a Shimadzu 6000 XRD spectrometer. Measurement conditions are 45 kV, 30 mA, and Cu Kα radiation with λ = 1.5406 Å at a scan range of 2θ = 5–80.

Attempts to record ¹H and ¹³C NMR spectra of the [Pd(H₂L)₂] complex in DMSO-d₆ and DMF-d₇ were unsuccessful due to the Chemical Shift Anisotropy (CSA) effect.

### Biological evaluation

#### *In Vitro Cytotoxicity Assay*

The cell lines used in this study were obtained from the American Type Culture Collection (ATCC, NY, USA). The MTT assay was used to assess the cytotoxic effects of [Pd(H_2_L)_2_] and violuric acid (VA) on three human cancer cell lines (liver carcinoma (HepG-2), colon carcinoma (HCT-116)), and breast adenocarcinoma (MDA-MB-231) as well as the normal amniotic cells (WISH cell line)), which served as a normal cell control, after a 72-hour incubation period. These results were compared with cisplatin (Cis), a standard anticancer drug. The assay procedure is described in the previous study^35^.

#### Apoptosis/Necrosis detection by flow cytometry

To evaluate the cell death mode (apoptosis/necrosis), treated HepG-2 cells were incubated with either half IC_50_ and IC_50_ of [Pd(H_2_L)_2_] complex and then maintained in a CO_2_ incubator for 24 h. Next, treated/untreated cells were placed in a cold PBS tube; then centrifuged in a cooling centrifuge for 15 min at 2000 rpm. After discarding the supernatants, the pellet was stained with the annexin V flowcytometry kit, then analysed with the BD Accuri C6 flow cytometer from BD Biosciences^35^.

#### Western blot analysis of p53 and caspase-3

The P53 and caspase 3 protein expression analysis was determined as listed in the previous study^36^. HepG-2 cells were treated with half the IC_50_ of [Pd(H_2_L)_2_] complex for 24 h. Afterward, cells were lysed, and the protein concentrations were measured. Equal amounts of protein were loaded into an SDS gel for separation by electrophoresis. P53 and caspase 3 proteins were detected using western blot analysis with specific antibodies: Anti-p53 (ab131442, abcam) and Anti-Caspase-3 (ab4051, abcam). The bands were treated with a chemiluminescent ECL substrate and imaged with a Chemi Doc imager (Biorad, USA). P53 and caspase 3 protein levels were quantified according to the recorded intensities of the emerged band (Image Lab Bio-Rad)^36^.

DNA/HSA-binding studies, viscosity measurements, and the method of DFT calculations are contained in Supplementary Materials S1.

### Statistical analysis

Data were shown as mean ± SEM. Minitab 18 Software, LLC, was used in statistical analysis. Groups were analyzed using a one-way ANOVA, and Tukey’s post hoc test was applied for multiple comparisons.

## Results and discussions

### Structural features and coordination mode of violuric acid

**Scheme 1:**
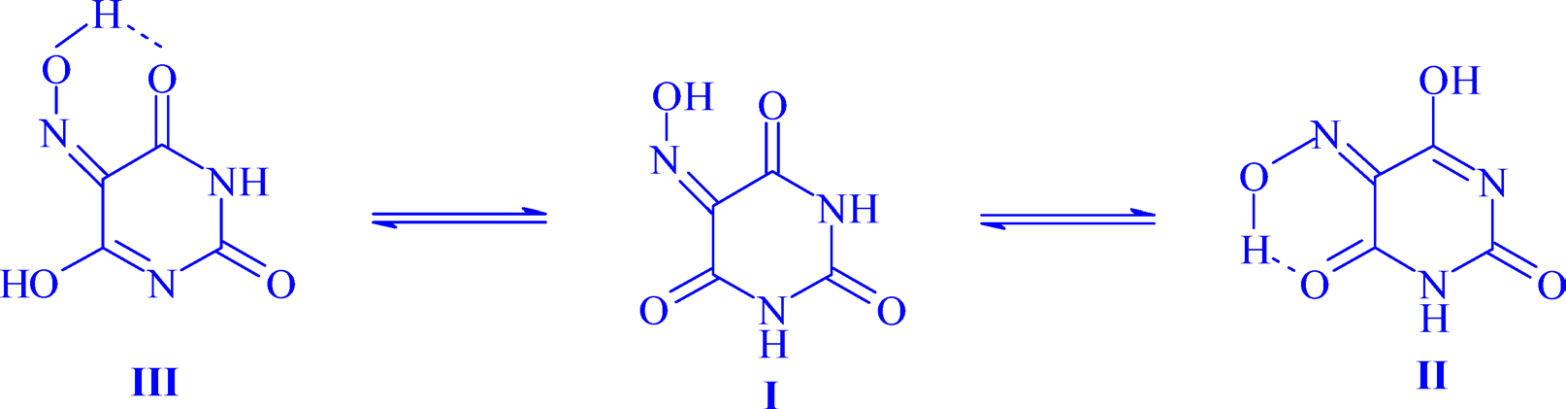
Possible tautomeric equilibrium of the parent violuric acid **I**.

Violuric acid, systematically named as 6-Hydroxy-5-nitroso-1 H-pyrimidine-2,4-dione (H₃L), is a heterocyclic compound based on a pyrimidine framework. It functions as a tribasic acid, exhibiting three distinct acid dissociation constants with pK_a_ values of 4.56, 9.60, and 13.10^28,37^. The molecular architecture of violuric acid (designated as **I** in Scheme 1) supports the existence of multiple tautomeric structures. At ambient temperature, theoretical studies indicate the possibility of up to ten tautomeric forms^38^. PM3 semi-empirical computational analyses reveal that the energy difference between the most stable and the second most stable tautomer is relatively small, approximately 1.59 kcal mol^‒1^[38]. Among these, tautomeric forms **II** and **III** (Scheme 1) are of particular interest due to their potential utility as chelating agents in coordination chemistry.

Although the violuric acid molecule (H₃L) possesses three ionizable protons, it frequently exhibits monobasic behavior in coordination reactions. Prior research has established that the violurate anion typically coordinates to metal centers in a bidentate fashion. This coordination mode involves the deprotonated oximato oxygen atom and the adjacent carbonyl oxygen atom derived from tautomeric forms **II** or **III**^19,20^.

**Scheme 2:**
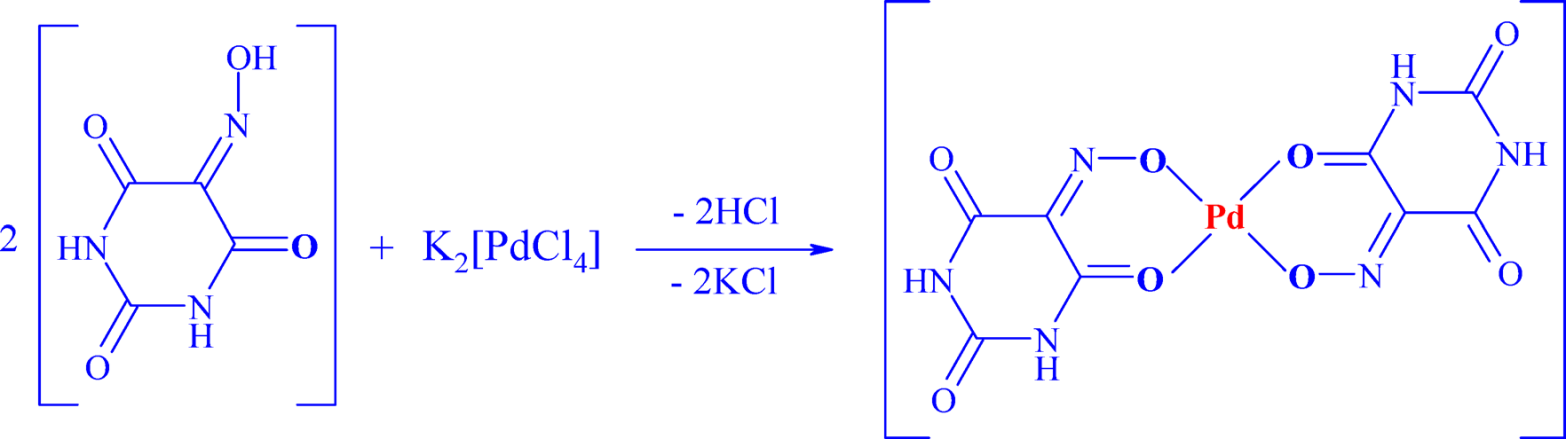
Illustration of the synthetic pathway of the [Pd(H₂L)₂] complex.

The current study involves the reaction of violuric acid with the palladium(II) salt, K_2_[PdCl_4_], at a 2:1 ligand-to-metal stoichiometric ratio in an aqueous-ethanolic solution, yielding a binary complex of the general formula [PdH_2_L₂] (Scheme 2). The molecular composition of the isolated complex was corroborated by elemental analysis data (Table 1). In the solid state, the palladium(II) violurate complex forms orange microcrystals and demonstrates notable stability under standard atmospheric conditions. Its solubility is limited, dissolving appreciably only in polar aprotic solvents such as dimethyl formamide (DMF) and dimethyl sulfoxide (DMSO).

Further insight into the nature of this complex was gained through conductivity measurements. Molar conductivity measurements of 0.001 M solution of the palladium(II) violurate complex in DMF yield a value of 15.55 Ω^‒1^ cm² mol^‒1^, indicating its non-electrolytic nature^39^. This supports the proposed coordination mechanism, wherein chloride anions are eliminated as HCl upon deprotonation of the ionizable acidic proton of the violuric acid ligand during complex formation. Consequently, these findings reinforce the role of violuric acid as a mono-anionic, bidentate chelating ligand, consistent with earlier reports in related systems^18–20^.

This close match between the experimental and theoretical values of elemental analysis data in Table 1 validates the proposed stoichiometry and purity of the synthesized palladium(II) violurate chelate. In the same regard, the mass spectrometry analysis recorded a molecular ion peak at m/z 417.57, which corresponds well to the calculated molar mass of the proposed molecular formula (C_8_H_4_N_6_O_8_Pd), confirming the formation of the intended mononuclear complex.

In summary, the collective analytical data spanning color, mass, conductivity, and elemental composition consistently support the successful synthesis and formation of the neutral, bis-chelate palladium(II) complex, [Pd(H₂L)₂].

**Table 1:** Elemental analysis, m/z, molar conductance, color of bis-chelate Pd(II) complex.

Complex	Color	m/z	Λ_M_ Ω^−1^ cm^2^ mol^− 1^	Found (Calcd.)			
				%C	%H	%*N*	%Pd
[Pd(H_2_L)_2_] C_8_H_4_N_6_O_8_Pd	Orange	417.57	15.55	22.95 (22.94)	0.96 (0.95)	19.75 (20.08)	25.50 (25.42)

Attempts to record ¹H and ¹³C NMR spectra of the [Pd(H₂L)₂] complex in DMSO-d₆ and DMF-d₇ were unsuccessful due to Chemical Shift Anisotropy (CSA) effect. This effect is well-documented in the literature for heavy metal complexes, even diamagnetic ones. The consequence is extreme signal broadening. The ¹H and ¹³C NMR signals become so broad that they merge into the baseline of the spectrum, making them impossible to detect or distinguish from background noise. Therefore, the complex was characterized using alternative techniques that have proven more informative for this system, including elemental analysis, mass spectrometry, thermal analysis, FTIR, UV-Vis, and PXRD combined with the Rietveld refinement approach, in addition to geometry optimization by DFT calculations.

### Mass spectrometry of [Pd(H₂L)₂

The spectrum of electro spray ionization mass spectrometry (ESI-MS) presented in Figure S2 provides key insights into the fragmentation pattern and stability of the synthesized palladium(II) violurate complex. The positive ion mode spectrum displays a prominent peak at m/z 417.57, corresponding to the molecular ion [PdC₈H₄N₆O₈]⁺. This signal confirms the formation of the intact complex and serves as a primary identifier for its molecular composition.

The fragmentation pattern observed in both positive and negative modes reveal a systematic degradation of the violurate ligand coordinated to the Pd(II) center. The successive losses of small neutral molecules and radicals are indicative of the complex’s stability under ionization conditions. Key fragments, such as [C₄H₂N₃O₄]⁺ (m/z 156.15), are likely derived from the violurate moiety following de-coordination or reductive elimination from the metal center. This fragment, along with subsequent breakdown products like [C₃H₂N₂O₃]⁺ (m/z 114.02) and [C₂H₂N₂O₂]⁺ (m/z 86.01), suggests a stepwise decomposition pathway involving the loss of CO, CN, and NO units, which is characteristic of aromatic N-heterocyclic carboxylate systems.

Furthermore, the detection of smaller inorganic and organic fragments, including [NO₂]⁺ (m/z 46.01), [C₂H₂NO₂]⁺ (m/z 72.01), and [‒N = O‒H]⁺ (m/z 31.01), supports the proposed structure of the ligand, confirming the presence of nitro and hydroxamate-like functional groups within the coordination sphere. The appearance of [C₂H₂N]⁺ (m/z 38.01) may arise from further rearrangement and cleavage of the heterocyclic core.

In conclusion, the mass spectral data are consistent with the formation of the target Pd(II)-violurate complex. The sequential fragmentation pattern not only validates the molecular integrity of the complex but also provides a fingerprint for its identification. The observed fragments align with the expected behavior of violurate-based metal complexes, underscoring the ligand’s coordination mode and its stability during mass spectrometric analysis. This analytical evidence corroborates the successful synthesis and structural assignment of the complex, which is relevant for its potential applications in catalysis or materials science.

### Thermal decomposition analysis (TGA/DTA)

Thermal analysis has emerged as an indispensable tool in the structural and compositional characterization of metal complexes, offering unique insights that complement spectroscopic and diffraction techniques. The thermal decomposition behavior of the [Pd(H₂L)₂] complex was investigated using simultaneous thermogravimetric (TGA) and differential thermal analysis (DTA). The thermogram in Figure S3 and data, summarized in Table 2, reveal a multi-step decomposition process leading to the eventual formation of metallic palladium.

The decomposition occurs in three distinct stages. The first stage, between 220 and 320 °C, is characterized by an endothermic DTA peak at 280 °C and a mass loss of 41.00% (calcd. 40.99%). This step corresponds to the partial decomposition of the coordinated violurate (H₂L) ligands, forming an intermediate species. The excellent agreement between observed and calculated mass loss confirms the stoichiometry of this initial degradation.

The second stage, spanning 320 to 400 °C, involves more complex thermal events. The DTA profile shows an exothermic doublet at 330 and 395 °C. The associated mass loss of 15.00% (calcd. 14.90%) aligns with the formation of a further degraded intermediate.

The final stage, from 400 °C to 1010 °C, leads to the complete removal of all remaining organic material. This is evidenced by two exothermic DTA peaks at 490 °C and 610 °C, alongside an endothermic peak at 950 °C. The total mass loss in this high-temperature region is 18.50% (calcd. 18.63%), culminating in the formation of pure palladium metal as the final residue. The calculated palladium content in the final residue is 25.41%, which is consistent with the theoretical value derived from the initial complex’s formula weight.

In conclusion, the TGA/DTA analysis demonstrates that the thermal decomposition of [Pd(H_2_L)_2_] proceeds through well-defined intermediate stages before yielding elemental palladium. The correlation between mass losses and proposed intermediate compositions is strong, and the DTA signals provide insight into the endothermic (e.g., ligand loss, decomposition) and exothermic (e.g., oxidation, combustion) nature of each transition.

**Table 2:** TGA and DTA analysis data of [Pd(H_2_L)_2_] complex.

Complex	Temperature °C	DTA °C	% Weight loss Found (Calcd.)	Species formed
[Pd(H_2_L)_2_]	220–320 320–400 400–1010	240* (330,395)**, (490, 610)**, 950*	41.00(40.99) 15.00(14.90) 18.50(18.63)	Intermediate residue Intermediate residue Pd

*Endothermic peak, **Exothermic peak.

### FTIR spectroscopy of violuric acid and [Pd(H_2_L)₂

Fourier-transform infrared (FTIR) spectroscopy was used to study the coordination behavior of violuric acid upon chelation with Pd(II) ions. The corresponding spectra are provided in the Supporting Information (Figures S4 and S5). Table 3 summarizes the characteristic vibrational frequencies of the free violuric acid ligand. Key absorption bands are observed at 3450 cm^‒1^(broad), 1760 cm^‒1^(strong), and 1595 cm^‒1^(strong), which are assigned to ν(OH), ν(C = O), and ν(C = N) stretching vibrations of the oximato moiety, respectively^19,40^. The broadened profile and specific frequency of the ν(OH) band suggest the presence of intramolecular hydrogen bonding within the free violuric acid molecule^19,41^.

The most notable change upon coordination is the complete disappearance of the broad ν(OH) band at 3450 cm^‒1^ observed for free violuric acid. This clearly indicates deprotonation of the hydroxyl group during complexation, confirming that the ligand coordinates to palladium in its monoanionic form (violurate)^41^.

Shifts in the carbonyl stretching frequency are highly informative. The ν(C = O) band shifts from 1760 cm^‒1^ in the free ligand to 1725 cm^‒1^ in the [Pd(H₂L)₂] complex. This shift to lower wavenumber is consistent with a decrease in C = O bond order, resulting from delocalization of the oxygen lone-pair density into the metal–oxygen bond upon coordination^42^.This confirms the involvement of the carbonyl oxygen atom in bonding to the Pd(II) center^43^.

The position of the ν(C = N) band remains stable at 1595 cm^‒1^ in both spectra, suggesting that the imine nitrogen does not participate directly in coordination to the metal ion in this complex, or that its bonding environment remains largely unchanged^42,44^.

A downward shift is also observed for the ν(N–O) stretching vibration, from 1240 cm^‒1^ to 1200 cm^‒1^. This shift can be attributed to the altered electron distribution within the ligand framework following deprotonation and coordination, which weakens the N–O bond^19,45^.

Finally, a new low-frequency band appears at 485 cm^‒1^ in the spectrum of the complex, which is absent in that of the free ligand. This band is assigned to the ν(O–Pd) stretching vibration and is characteristic of the newly formed metal–oxygen coordinate bond, providing direct evidence for the successful formation of the Pd(II) chelate^46^.

In conclusion, the FTIR spectral analysis strongly supports the formation of a Pd(II) complex in which the monoanionic violurate ligand coordinates to the metal center primarily through the oxygen atom of the deprotonated hydroxyl group (enolic oxygen) and likely the adjacent carbonyl oxygen, forming a stable chelate ring^45,46^. The spectral changes, specifically the disappearance of ν(OH), the shift in ν(C = O), and the emergence of the ν(O–M) band, collectively confirm the proposed coordination mode. Accordingly, the Pd(II) center is coordinated via four coordinate covalent (dative) bonds to two O, O-bidentate violurate ligands, forming a stable square-planar geometry.

Together with mass spectrometry, molar conductivity, and thermal analysis data, the FTIR results consistently support the successful synthesis and proposed molecular structure of the neutral, bis-chelate palladium(II) violurate complex, [Pd(H₂L)₂], shown in Scheme 2.

**Table 3:** FTIR spectral data (cm^− 1^) of violuric acid and its Pd(II) chelate.

Compound	υ(OH)	υ(C = O)	υ(C = *N*)	υ(*N*-O)	υ(O-M)
Free H_3_L*	3450	1760	1595	1240	-
[Pd(H_2_L)_2_]	-	1725	1595	1200	485

*Violuric acid.

### UV-visible absorption spectroscopy

The electronic absorption spectra of violuric acid (VA) and its Pd(II) complex, [Pd(H_2_L)_2_], provide significant insights into the electronic transitions and coordination environment upon complexation with palladium(II) ion. The spectral features (Figures S6 and S7), summarized in Table S8, reveal pronounced shifts and the emergence of new bands, which can be attributed to changes in molecular symmetry, ligand field effects, and charge distribution^46^.

Violuric acid exhibits two main absorption bands: a high-energy band at 262 nm assigned to π → π* transitions within the pyrimidine ring, and a lower-energy band at 340 nm attributable to n → π* transitions of the carbonyl and oxime groups^47^. The Pd(II) complex shows red-shifted bands at 270 and 355 nm, assignable to (π→π*) and 340 nm (n→π*) transitions, respectively.

Upon coordination with Pd(II), two new low - energy absorption bands appear at 562 nm and 730 nm. These are attributed to d-d transitions within the Pd(II) center, specifically from the ground state (^1^*A*_1g_) to excited states (^1^*A*_2g_) and (^1^*B*_1g_), respectively, in a square-planar geometry^48,49^. The presence of these bands confirms the formation of a coordination complex and provides evidence of the ligand field splitting induced by the violurate ligands. The ratio (ν_1_/ν_2_) (approximately 0.77) is consistent with values expected for square-planar Pd(II) complexes, supporting the proposed geometry^49–51^.

The π → π* transition in the complex is observed at 262 nm, showing a slight red shift compared to the free ligand (270 nm). This shift suggests a reduction in π-conjugation or increased rigidity upon coordination, possibly due to changes in planarity or electron withdrawal by the metal center. In the same regard, the n → π* transition undergoes a red shift from 340 nm in free violuric acid to 355 nm in [Pd(H_2_L)_2_], indicating enhanced stabilization of the non-bonding orbitals or decreased electron density on the hetero atoms due to coordination^51–53^.

While not explicitly assigned in the table, the low-energy region (562–730 nm) may also involve ligand-to-metal charge transfer (LMCT) contributions, given the electron-rich nature of the violurate ligand and the relatively high oxidation state of Pd(II). Such transitions often overlap with d-d bands and can affect the overall absorption profile^47^.

The electronic absorption spectra of violuric acid and its Pd(II) complex highlight significant alterations upon coordination. The appearance of d-d transitions confirms complex formation and provides structural insights into the ligand field environment. Shifts in the π → π* and n → π* bands reflect changes in electronic distribution and molecular geometry. These findings contribute to a deeper understanding of violurate-based coordination chemistry and may inform further studies on related systems for applications in catalysis, sensing, or bioinorganic chemistry.

### Crystal structure and DFT analysis

Although single crystals suitable for X-ray diffraction could not be obtained despite repeated attempts, the combination of PXRD with Rietveld refinement and DFT calculations provides a reliable and validated structural model, as previously reported for analogous systems^54–57^. In this work, the PXRD pattern of the [Pd(H₂L)₂] complex (Fig. S9) was indexed and refined using the Expo 2014 software suite via the Rietveld method^58^. The proposed structural model was deposited and validated by the Cambridge Crystallographic Data Centre (CCDC), receiving deposition number 2,484,420 and (DOI:10.5517/ccdc.csd.cc2pd7ly). The experimental PXRD profile (Fig. S10) demonstrates an excellent match with the simulated pattern derived from the computational model. Successful structure solutions and Rietveld refinement were accomplished, resulting in satisfactory reliability factors.

The calculated density (ρ = 1.728 g·cm^‒3^ was derived from the crystallographic equation ρ = (Z × M)/(NA × V), where Z = 2 (number of formula units per unit cell), M = 418.574 g·mol^‒1^(molecular weight), NA = 6.022 × 10^23^ mol^‒1^(Avogadro’s number), and V = 804.3 Å^3^ (unit cell volume). The excellent internal consistency between these parameters confirms both the molecular formula and the unit cell content.

#### Crystallographic data

The crystallographic data (Table 4) for the Pd(II) complex, [Pd(H₂L)₂], were successfully obtained from X-ray diffraction analysis and refined using the Rietveld method. The complex crystallizes in the triclinic crystal system with the centrosymmetric space group *P* − 1 (space group number 2), indicating the presence of an inversion center within the unit cell. The unit cell parameters are a = 19.256 Å, b = 6.982 Å, c = 6.347 Å, α = 108.62°, β = 94.44°, and γ = 84.77°, resulting in a cell volume of 804.3 Å^3^. The calculated density of 1.728 g·cm^‒3^ is consistent with the molecular formula [C₈H₄N₆O₈Pd] and a formula weight of 418.574 g·mol^‒1^.

The quality of the structural refinement is reflected in the reliability factors obtained from the Rietveld analysis. The profile residuals Rp = 12.510 and Rwp = 16.011, while the Bragg factor R-Bragg = 23.200. A goodness-of-fit (χ²) value of 1.48 indicates a satisfactory agreement between the observed and calculated diffraction patterns. The average crystallite size, estimated from the diffraction data, is approximately 45.62 nm. These crystallographic parameters provide a solid foundation for understanding the molecular packing and solid-state structure of the complex, which is essential for correlating its structure with its physicochemical properties and potential applications in catalysis or materials science. The centrosymmetric nature of the space group may imply specific symmetrical constraints on the molecular geometry of the complex in the solid state.

**Table 4:** Crystallographic information of Pd(II) violurate complex.

**Crystallographic property**	[Pd(H_2_L)_2_] complex
Empirical formula	C_8_H_4_N_6_O_8_Pd
Formula weight	418.574
T (K)	277.55
l (Å)	1.5406
Crystal system	Triclinic
Space group	P −1
Centro symmetry	Centric
Space Group Number	2
Z	2
Multiplicity	2
Bravais Lattice	P
Lattice Symbol	tP
Crystallite size (nm)	45.62
**Unit cell dimensions:**	
*a* (Å),	19.256,
*b* (Å),	6.982,
*c* (Å)	6.347
*a* (°),	108.62,
*b* (°),	94.44,
*g* (°)	84.77
Cell volume (Å^3^)	804.3
Volume per atom (Å^3^)	17.484
Calculated density (g/cm^3^)	1.728
q range for data collection (°)	15.000–43.000
Total reflection	140
**Rietveld results:**	
Rp	12.51
Rwp	16.011
R-Bragg	23.2
Goodness of fit	1.48
Deposition number	CCDC2484420, DOI:10.5517/ccdc.csd.cc2pd7ly

#### Hydrogen bonding and molecular packing

**Fig. 1:**
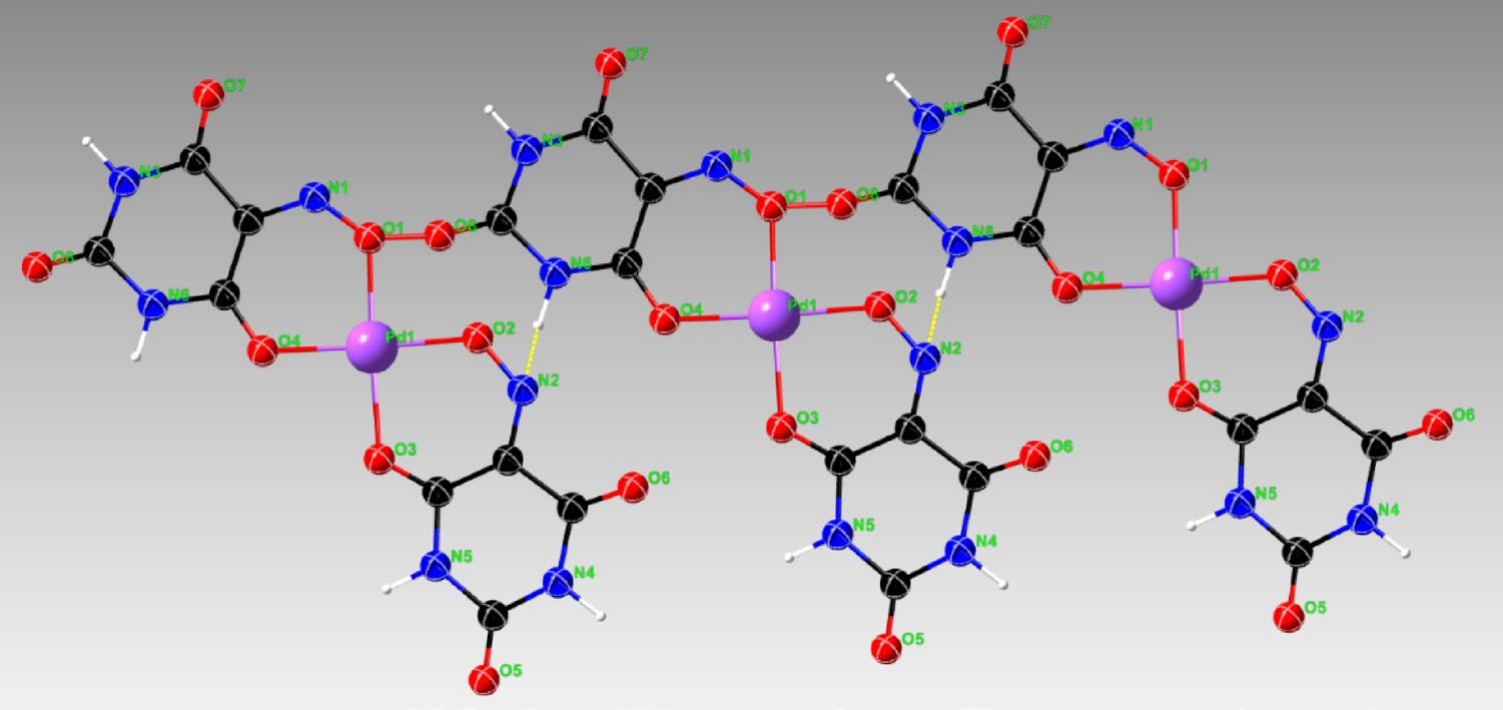
Hydrogen bonding pattern in the crystal structure of [Pd(H_2_L)_2_] complex.

**Fig. 2:**
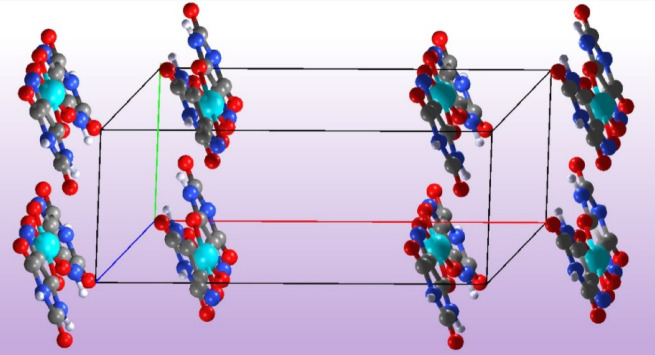
Molecular packing pattern in the crystal structure of [Pd(H_2_L)_2_] complex.

The presented data in Table 5 and the accompanying Figs. 1 and 2 provide crucial structural insights into the hydrogen bonding network within the crystal structure of the palladium complex [Pd(H_2_L)_2_], where H_2_L is mono anionic violurate ligand.

Table 5 lists the fractional coordinates (×10⁴) and isotropic displacement parameters, U(eq) (×10³ Å²), for four hydrogen atoms (H1–H4) associated with the structure. The coordinates precisely locate these atoms within the unit cell. Notably, all four hydrogen atoms exhibit identical U(eq) values of 45.6, suggesting a relatively uniform thermal motion or similar chemical environment for these specific protons in the crystal lattice at the measured temperature. This uniformity may indicate that these atoms are involved in comparable intermolecular interactions, such as hydrogen bonds of similar strength, contributing to the overall lattice stability.

The provided molecular diagram depicts the coordination environment of the palladium center and the structure of the coordinated violurate ligands. More importantly, it visually represents the key intermolecular hydrogen-bonding interactions (shown as dashed lines) that extend the molecular structure into a three-dimensional network. The figure allows for the identification of potential donor and acceptor atoms involved in these bonds, which are critical for understanding the packing arrangement and stabilization of the crystal.

The hydrogen atoms listed in Table 5 (H1–H4) are likely those involved in the hydrogen-bonding interactions illustrated in the figure. The combination of quantitative positional data from the table and qualitative connectivity information from the figure enables a comprehensive description of the supramolecular architecture. Hydrogen bonds in this system typically involve the NH or OH groups of the violuric acid ligands as donors and carbonyl oxygen atoms or other suitable acceptors from adjacent molecules. These interactions are paramount for directing the crystal packing, influencing the material’s physical properties, and contributing to the overall stability of the solid-state structure.

In summary, the structural data support the proposal that the [Pd(H_2_L)_2_] complex forms a well-defined hydrogen-bonded network in the solid state. The precise atomic coordinates facilitate the calculation of specific hydrogen bond distances (D–H···A) and angles, which are essential for quantifying interaction strengths. The consistent thermal parameters for the hydrogen atoms support a model of a robust and ordered crystalline lattice stabilized by these intermolecular forces. This analysis underscores the role of mono valent violurate anion (H_2_L^‒^) not only as a coordinating ligand but also as an active participant in building a supramolecular framework through its hydrogen-bonding capabilities. The present data support the proposed active role of hydrogen bonding in the crystal engineering of the [Pd(H_2_L)_2_] complex. The uniform displacement parameters and the visualized network suggest a stable and ordered arrangement sustained by these non-covalent forces.

**Table 5:** Hydrogen bond parameters of [Pd(H_2_L)_2_] complex.

Hydrogen Atom Coordinates (Å×10^4^) and Isotropic Displacement Parameters (Å^2^ × 10^3^)				
**Atom**	***x***	***y***	***z***	**U(eq)**
H1	6743	6674	−6968	45.6
H2	9268	10,973	14,666	45.6
H3	9151	6468	8428	45.6
H4	7919	4369	−2141	45.6

#### Coordination geometry (τ₄ parameter)

**Fig. 3:**
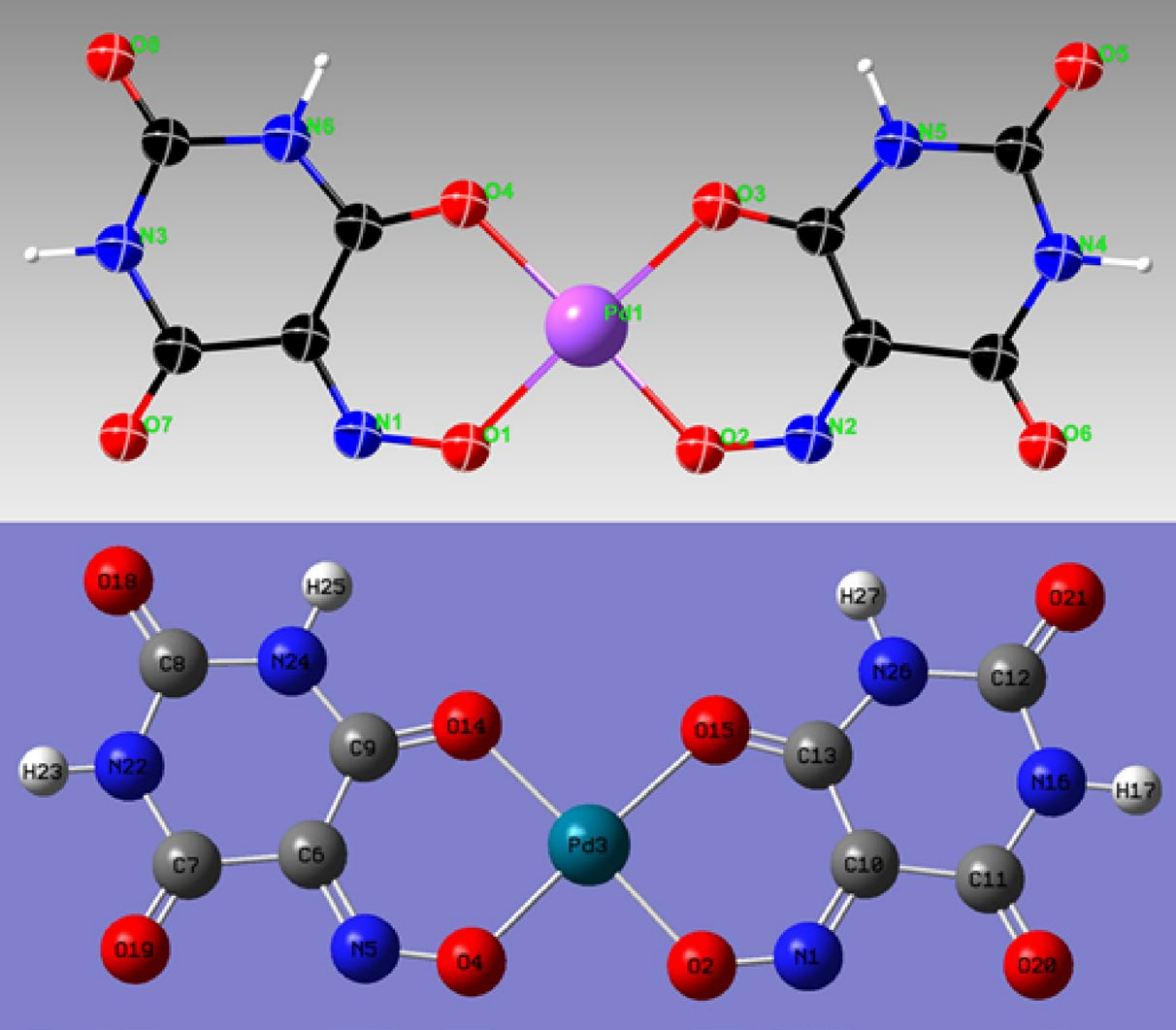
Optimal geometry of the [Pd(H_2_L)_2_] compound according to powder X-ray diffraction (PXRD) data processing (top) and density functional theory (DFT) calculations (bottom).

The stereochemistry of the [Pd(H₂L)₂] complex was investigated by examining key bond angles obtained from both PXRD and DFT calculations, as detailed in Table 6. The τ₄ parameter, which quantifies the geometry of four-coordinate complexes, was calculated to be 0.04. This value is characteristic of an ideal square-planar geometry^59^. This conclusion is further supported by the close agreement between experimental and computed bond angles, with no significant deviation toward tetrahedral or other geometries, as depicted in Fig. 3.

The bond angles around the palladium (Pd1) center in the complex, as determined by processing the powder X-ray diffraction (PXRD) data by Expo 2014 software and density functional theory (DFT) calculations, are summarized in Table 6. The τ₄ parameter of 0.04 confirms a nearly ideal square planar geometry around the Pd(II) of [Pd(H_2_L)_2_] complex, with only minor deviations.

**Table 6:** The most relevant bond angle (ᶱ) around Pd(II) core of the [Pd(H_2_L)_2_] complex.

Geometrical index of Pd1 centre (τ_4_ = 0.04) Square planar geometry			
Bond type	XRD	DFT	Difference
O1- Pd1- O2	87.26	85.76096	1.49904
O1- Pd1- O3	177.46	177.40148	0.05852
O1- Pd1- O4	90.08	91.64018	1.56018
O2- Pd1- O3	90.21	91.64054	1.43054
O2- Pd1- O4	177.33	177.40114	0.07114
O3- Pd1- O4	92.46	90.95833	1.50167

A comparison between the experimental (PXRD) and theoretical (DFT) bond angles reveals generally excellent agreement, with absolute differences ranging from 0.058° to 1.560°. The most significant discrepancies are observed for the O1–Pd1–O4 and O2–Pd1–O3 angles (≈ 1.56° and ≈ 1.43°, respectively), while the trans angles (O1–Pd1–O3 and O2–Pd1–O4) show remarkable consistency, differing by less than 0.072°. This close match for the *trans* angles underscores the accuracy of the DFT method in reproducing the core ligand arrangement in the square plane.

The minor deviations in the adjacent (*cis*) angles likely arise from a combination of factors, including crystal packing forces and intermolecular interactions in the solid state (captured by XRD but not fully in the gas-phase DFT model), as well as the inherent approximations of the chosen DFT functional and basis set. Nevertheless, the overall congruence between theory and experiment validates the employed computational methodology and supports the assigned molecular structure.

These results are consistent with the expected electronic configuration and coordination environment for a Pd(II) center, which typically adopts a d⁸ square planar geometry to minimize ligand–ligand repulsion and achieve optimal orbital overlap. The small τ₄ value and the angle set confirm minimal distortion from ideal geometry, which is relevant for understanding the complex’s reactivity, spectroscopic properties, and potential applications in catalysis or materials science.

In conclusion, the structural data from both XRD and DFT affirm a stable square planar coordination sphere around the Pd(II) center, with computational results providing reliable support for the experimentally observed geometry as shown in Fig. 3. The persistence of square planar geometry across both analyses reinforces the structural assignment and provides a robust foundation for further electronic property investigations of this [Pd(H_2_L)_2_] complex.

#### Bond length analysis

The selected bond distances for the Pd(II) complex, as determined by processing powder X-ray diffraction (PXRD) data and density functional theory (DFT) calculations, are presented in Table 7. The structural analysis centers on the Pd1 atom (Fig. 3), which exhibits a τ₄ parameter of 0.04, confirming a near-perfect square planar coordination geometry, characteristic of d⁸ Pd(II) complexes.

A comparison between the experimental (XRD) and theoretical (DFT) bond lengths reveals a consistent trend: the DFT-calculated distances are systematically shorter than their XRD counterparts. The differences range from 0.0552 Å for the Pd1-O2 bond to 0.11227 Å for the Pd1-O3 bond. The minimal deviation from ideal square planar geometry (τ₄ ≈ 0) is supported by both datasets, confirming the expected coordination environment for this Pd(II) center.

It is important to note the inherent differences between the two methods when comparing bond lengths and angles. The PXRD data represents the time- and space-averaged structure within the crystal lattice at 277.55 K, influenced by crystal packing forces, thermal motion, and intermolecular interactions (e.g., hydrogen bonding). In contrast, DFT calculations model an isolated molecule in the gas phase at 0 K, devoid of solid-state effects. Therefore, systematic differences, particularly the consistently shorter DFT bond lengths (by 0.055–0.112 Å), are expected and well-documented in the literature. These discrepancies arise from the absence of harmonic vibrations, environmental effects, and limitations in the chosen functional/basis set. Despite these systematic deviations, the relative trends in bond distances are well-preserved between the two methods, validating the DFT approach for capturing the essential electronic and geometric features of the complex.

The bond distances from PXRD were obtained from the Rietveld-refined structural model (CCDC 2484420). Following convergence of the refinement (Rp = 12.510, Rwp = 16.011, χ² = 1.48), the fractional atomic coordinates were used to calculate interatomic distances between the Pd1 center and coordinated oxygen atoms using the standard distance formula: d = √[(Δx·a)² + (Δy·b)² + (Δz·c)² + 2ΔxΔy·ab·cosγ + 2ΔxΔz·ac·cosβ + 2ΔyΔz·bc·cosα]. The estimated standard deviations for bond distances are approximately ± 0.005–0.01 Å based on the refinement quality.

In conclusion, the agreement in structural trends between experimental and calculated data supports the proposed molecular geometry. The observed absolute differences in bond lengths are within the range reported for such comparative analysis and may reflect differences between solid-state measurements and gas-phase theoretical models. These findings highlight the complementary roles of XRD for empirical solid-state structure elucidation and DFT for understanding the intrinsic electronic structure of the molecule.

**Table 7:** Selected bond lengths of [Pd(H_2_L)_2_] complex.

Bond distance (Ǻ)				
Bond Type	XRD	DFT	Difference	
Pd1- O1	2.11716	2.00976	0.10740	
Pd1- O2	2.06494	2.00974	0.05520	
Pd1- O3	2.17789	2.06562	0.11227	
Pd1- O4	2.12408	2.06560	0.05848	

#### DFT calculations and electronic structure

##### Optimized geometry

Density functional theory (DFT) calculations have emerged as an essential computational tool in inorganic chemistry, offering detailed predictions of the structural and electronic properties of metal complexes^60^. In this study, DFT was employed to perform a geometric optimization of the [Pd(H₂L)₂] complex. The computed bond lengths and angles (Tables 4 and 5) show remarkable consistency with the experimental PXRD data. This strong correlation validates the proposed structure for the [Pd(H₂L)₂] complex and is in line with reported data for analogous systems^34,61^. The collective analytical findings may be correlated to the formation of a neutral, mononuclear complex, where the Pd(II) center resides in a square-planar coordination sphere, chelated by two O, O-donor violurate ligands in a bis-bidentate fashion (Fig. 3)^62,63^.

##### Global reactivity descriptors

The computed global reactivity descriptors (Table S11, Figures S12 and S13) provide a quantitative framework for comparing the electronic structures and chemical behavior of violuric acid (H₃L) and its Pd(II) complex, [Pd(H₂L)₂]. The analysis reveals significant modifications in reactivity upon complexation^64^.

The energy of the lowest unoccupied molecular orbital (LUMO) increases from − 1.3382 eV in the free ligand to −1.99732 eV in the complex, indicating that the palladium complex possesses a significantly higher electron affinity and is more susceptible to nucleophilic attack^65^. Conversely, the energy of the highest occupied molecular orbital (HOMO) also rises from − 7.8555 eV to −7.4029 eV, suggesting a greater propensity for the complex to act as an electron donor, though it remains less pronounced than the change in LUMO energy.

The most notable change is observed in the HOMO-LUMO energy gap (ΔE), which decreases from 6.517 eV in the ligand to 5.406 eV in the complex. This substantial reduction in band gap is a strong indicator of the complex’s enhanced chemical reactivity and lower kinetic stability compared to the isolated ligand^66^. This is further corroborated by the chemical hardness (η) and softness (S) parameters. The complex exhibits lower hardness (2.703 vs. 3.259) and higher softness (0.185 vs. 0.153), characterizing it as a “softer” and more polarizable species according to Pearson’s Hard-Soft Acid-Base (HSAB) principle^67^.

The electronegativity (χ) is marginally higher for the complex (4.700 vs. 4.597), while its chemical potential (µ) becomes more negative (−4.700 vs. −4.597). This implies that the complex is slightly more electronegative and has a lower overall energy, contributing to its stabilization, yet its increased softness overrides this to promote reactivity^68^. The electrophilicity index (ω), which measures the energy lowering due to maximal electron flow from the environment, is significantly greater for the complex (4.087 vs. 3.242), identifying it as a superior electrophile. Correspondingly, the maximum electron transfer index (ΔN_max_) is higher for the complex (1.739 vs. 1.411), quantifying its increased capacity to accept electronic charge^69^.

In summary, the coordination of violuric acid to palladium(II) engenders a molecule with a distinctly more reactive profile. These computational insights elucidate the fundamental electronic changes induced by metal complexation and may support the proposal that [Pd(H_2_L)_2_] has potential in applications requiring electron-accepting properties, such as catalysis or biological interactions^70,71^.

##### Reactivity descriptors vs. DNA/HSA binding

Quantum chemical-derived global reactivity descriptors rationalize the binding affinities of violuric acid (VA) and its Pd(II) complex, [Pd(H₂L)₂], toward DNA and HSA (Tables 8, 9, 10 and 11, S11)^21,72,73^.

The HOMO–LUMO energy gap (ΔE) is smaller for [Pd(H₂L)₂] (5.406 eV) than for VA (6.517 eV), indicating higher reactivity and polarizability^74,75^. Consistently, the complex shows higher experimental binding constants (Kb) for DNA (1.694 × 10⁴ M^‒1^ vs. 1.325 × 10⁴ M^‒1^ and HSA (2.8 × 10⁵ M^‒1^ vs. 1.6 × 10⁴ M^‒1^^18,76^.

The electrophilicity index (ω) is larger for the complex (4.087 vs. 3.242), promoting electrostatic or charge-transfer interactions with electron-rich biomolecular sites^77^. This aligns with stronger fluorescence quenching (Kq ~ 10¹³–10¹⁴ M^‒1^ s^‒1^ and hyperchromism^9,78^.

The complex exhibits lower chemical hardness (η: 2.703 vs. 3.259) and higher softness (S: 0.185 vs. 0.153), enhancing polarizability and binding to soft nucleophilic sites (e.g., DNA bases, Trp residues). This is supported by more negative ΔG values for DNA binding (–24.12 vs. − 23.51 kJ/mol)^79^.

Maximum electron transfer number (ΔN_max_) is higher for the complex (1.739 vs. 1.411), favoring charge-transfer complex formation and explaining spectral shifts (bathochromic/hypsochromic, hyperchromism)^80^.

Overall, the complex’s lower ΔE, higher ω, greater softness, and higher ΔN_max_ correlate with enhanced DNA/HSA affinity, stronger quenching, and more spontaneous binding^81^. These quantum chemical descriptors complement experimental data, though they provide correlative rather than causative evidence^19,47^. Direct experimental validation remains necessary.

### Biomolecular interaction studies

#### DNA binding studies

##### UV-Vis absorption of DNA adducts

The UV-visible absorption spectral data presented in Table 8 provide significant insights into the binding interactions of free violuric acid and the [Pd(H₂L)₂] complex with DNA. Both compounds exhibit characteristic changes upon interaction with the biomolecule, indicative of a binding event.

A minor hypsochromic (blue) shift is observed for both systems: Δλ = 2 nm for free VA (from 262 nm to 260 nm) and Δλ = 5 nm for the [Pd(H₂L)₂] complex (from 270 nm to 265 nm). This shift suggests that the binding mode likely involves external contact or groove binding, as significant intercalation typically results in bathochromic (red) shifts due to strong π-π stacking interactions between the aromatic chromophore and DNA base pairs.

**Fig. 4:**
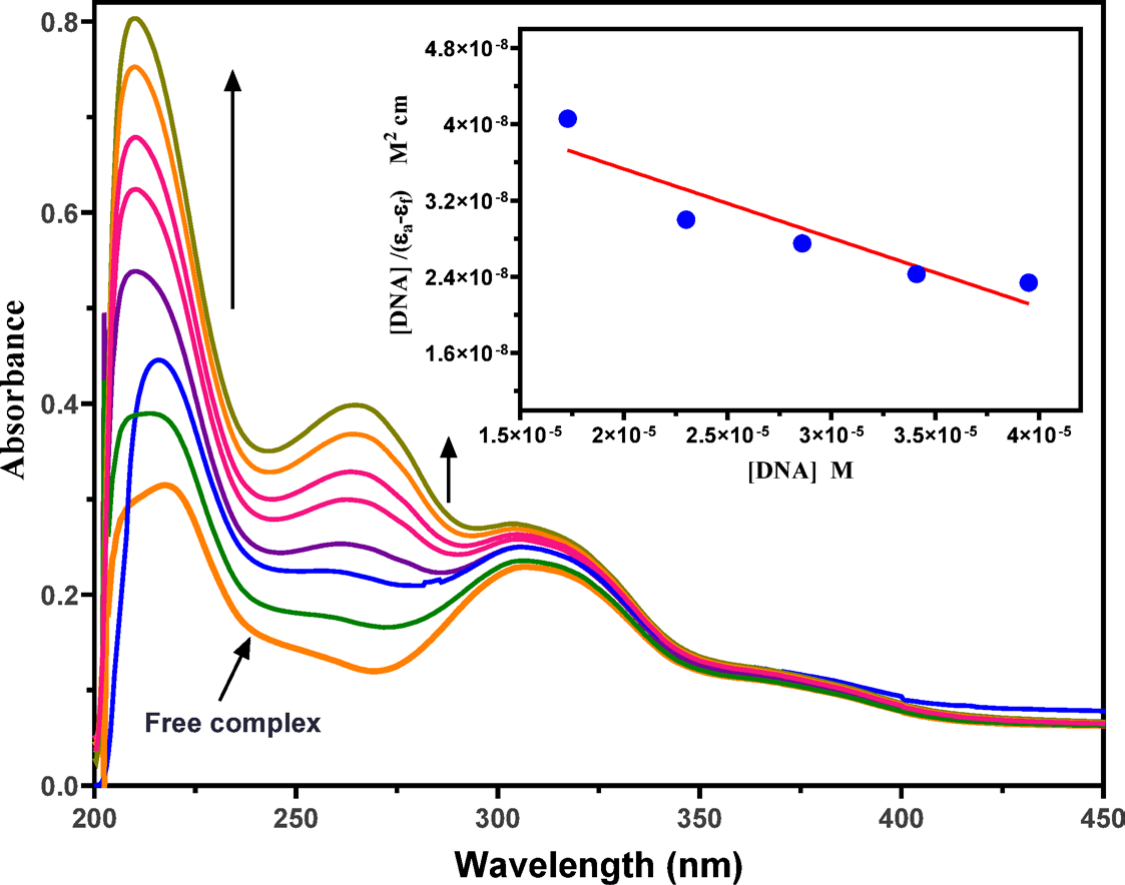
UV-visible absorption spectral profile of [Pd(H₂L)₂] binding with ct-DNA. Fixed concentration of the complex (20 µM) was titrated with increasing ct-DNA concentrations (0–10 µM) in Tris-HCl buffer (pH 7.4). Hyperchromism (47.11% increase at λ_max_ = 270 nm) with significant wavelength shift (5 nm) indicates surface binding or electrostatic interactions with the ct-DNA. The binding constant (K_b_ = 1.694 × 10^4^ M⁻¹) was calculated from the Benesi-Hildebrand plot (inset).

Notably, both interactions are characterized by hyperchromism, indicating an increase in absorption intensity upon DNA binding. The hyperchromic effect, quantified as 47.11% for free VA and 34.88% for the [Pd(H₂L)₂] complex, is often associated with changes in the DNA secondary structure or perturbations in the stacking of nitrogenous bases, potentially due to partial unwinding of the double helix or electrostatic interactions.

The binding affinity, quantified by the binding constant (K_b_), is of the order of 10^4^ M^‒1^ for both compounds (1.325 × 10^3^ M^‒1^ for VA and 1.694 × 10^4^ M^‒1^ for [Pd(H₂L)₂]). These values confirm a moderately strong and spontaneous interaction with DNA, as further evidenced by the negative Gibbs free energy changes (ΔG): −23.51 kJ mol^‒1^ for VA and − 24.12 kJ mol^‒1^ for the complex. The slightly more negative ΔG value for the palladium complex suggests a marginally more favorable binding process compared to the free ligand, which may be attributed to the coordinated metal center influencing the overall charge, geometry, and electronic properties of the molecule, potentially facilitating stronger electrostatic or coordinative interactions with the DNA phosphate backbone.

The spectral profiles illustrated in Figs. 4 and S14 visually corroborate these findings. The figures clearly demonstrate the hyperchromic effect and the slight blue shift in the absorption maxima for both [Pd(H₂L)₂] and violuric acid upon titration with DNA, providing direct graphical evidence of the formation of a DNA adduct.

Compared to reported nickel complexes such as [NiL₂L’]^82^ (K_b_ = 2.20 × 10⁵ mol^‒1^ dm³, hyperchromism 25.44%), [Ni(HL)₂]Cl₂^83^ (K_b_ = 3.6 × 10³ mol^‒1^ dm³, hypochromism), and NiL_2_^84^ (K_b_ = 2.24 × 10^3^ mol^‒1^ dm³, hypochromism). The palladium complex in this work exhibits moderate binding strength, superior to free VA and some Ni(II) complexes, though lower than [NiL₂L’]. The negative ΔG values confirm spontaneous binding for all compounds.

In conclusion, the UV-vis absorption studies suggested that both violuric acid and its palladium(II) complex interact with DNA, likely through a non-intercalative, such as groove or external binding mode, as indicated by hyperchromism and minor blue shifts; however, the exact binding mode was not directly confirmed. The palladium complex showed a comparable and slightly higher apparent binding affinity when compared to the ligand, indicating that metal coordination may influence DNA interaction properties. These findings indicate that the possibilities offered by the metal-based complexes can modulate DNA interactions under the studied conditions. This data may be relevant for the design of DNA-targeting agents.

**Table 8:** The UV-visible absorption spectral data for the binding interactions of the free VA and [Pd(H_2_L)_2_] complex with DNA.

Compound	λ_max_(nm) free	λ_max_(nm) bound	Δλ	Type of chromism	Chromism (%)^a^	(K_b_) mol^− 1^ dm^3^	ΔG kJ mol^− 1^	Ref.
Free VA	262	260	2	hyper	47.11	1.325 × 10^3^	−23.51	This work
[Pd(H_2_L)_2_]	270	265	5	hyper	34.88	1.694 × 10^4^	−24.12	This work
[NiL_2_L’]	270	269	1	hyper	25.44	2.20 × 10^5^	−30.47	^82^
[Ni(HL)_2_]Cl_2_	402	400	-	hypo	-	3.6 × 10^3^	−20.29	^83^
NiL_2_	320	320	-	hypo	-	2.24 × 10^3^	−19.11	^84^

^a^Chromism (%) = [(Abs _bound_ − Abs _free_)/Abs _free_].

##### Fluorescence quenching of DNA binding

The fluorescence quenching data presented in Table 9 provide significant insights into the binding interactions of the free ligand H₂L and its palladium(II) complex [Pd(H_2_L)_2_] with DNA. The analysis of the Stern-Volmer quenching constant (Ksv), the bimolecular quenching constant (K_q_), the binding constant (K_b_), the number of binding sites per nucleotide (n), and the Gibbs free energy change (ΔG) reveals pronounced differences between the two compounds, highlighting the impact of metal coordination on DNA-binding affinity and mechanism.

For the free ligand VA, the Ksv value of 7.732 × 10^3^ M^‒1^ and the corresponding K_q_ value of 7.732 × 10^11^ M^‒1^ s^‒1^, which exceeds the maximum diffusion-controlled quenching constant (~ 10^10^ M^‒1^ s^‒1^, suggest that the quenching mechanism is predominantly static^85^. This indicates the formation of a non-fluorescent ground-state complex between the ligand and DNA. The binding constant K_b_ is 1.836 × 10^3^ M^‒1^, with a binding site n of approximately 0.77, implying a binding stoichiometry of one ligand per nucleotide. The negative ΔG value of −18.62 kJ mol^‒1^ confirms a spontaneous binding process.

In contrast, the palladium complex [Pd(H_2_L)_2_] showed an apparent increase in DNA-binding affinity. The Ksv value increases to 1.327 × 10^5^ M^‒1^, and the K_q_ value (1.327 × 10^13^ M^‒1^ s^‒1^ is consistent with a static quenching mechanism^85^. Notably, the binding constant K_b_ reaches 2.870 × 10⁷ M^‒1^, which is approximately four orders of magnitude greater than that of the free ligand. This increase suggests a stronger interaction with DNA under the studied conditions. The binding site size *n* > 1 (1.503) may suggest the possibility of multiple binding modes or the involvement of more than one complex molecule per binding site. This increase is consistent with the interaction with DNA under the experimental conditions used. The more negative ΔG value compared to the free ligand indicates a more favorable Gibbs free energy change for the complex-DNA adduct formation *in vitro.*

**Fig. 5:**
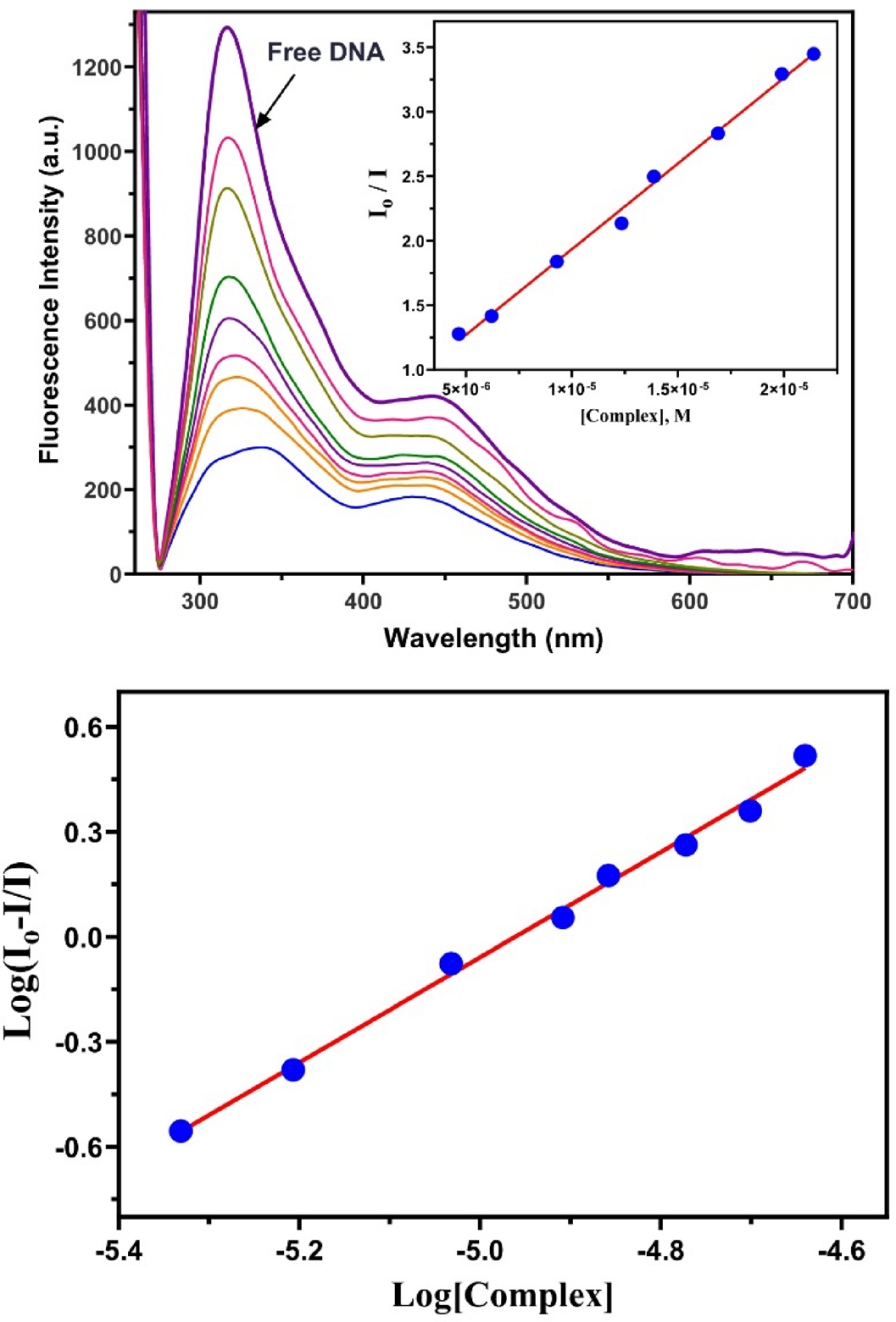
Fluorescence quenching spectral profile of [Pd(H₂L)₂] binding with DNA. Increasing concentrations of DNA (0–100 µM) were titrated against a fixed concentration of the complex (10 µM) in Tris-HCl buffer (pH 7.4, λ_max_ = 290 nm). The observed decrease in fluorescence intensity (quenching) with increasing DNA concentration indicates complex-DNA adduct formation. The inset shows the Stern-Volmer plot (F₀/F vs. [DNA]), where linearity confirms a static quenching mechanism. The high quenching constant (Ksv = 1.327 × 10⁵ M⁻¹) suggests a strong groove-binding interaction.

The excellent linear correlations (R² > 0.98) for both compounds validate the reliability of the fitted parameters. The observed enhancement in binding parameters may be attributed, in part, to the increased positive charge and planarity of the complex, which could facilitate electrostatic and groove-binding interactions with DNA. This pronounced increase in DNA affinity positions [Pd(H_2_L)_2_] as a far more potent candidate for applications requiring strong DNA interaction, such as in chemotherapeutic agents or molecular probes.

The fluorescence quenching spectral profiles (Figs. 5 and S15) visually support these quantitative findings. Both figures likely exhibit a characteristic decrease in fluorescence emission intensity with increasing DNA concentration, confirming the quenching phenomenon. The more pronounced quenching observed in the spectrum of [Pd(H₂L)₂] aligns perfectly with its significantly higher Ksv and K_b_ values.

Compared to reported nickel complexes, such as [NiL₂L’] (K_b_ = 1.410 × 10⁴ mol^‒1^ dm^3^, Ksv = 9.928 × 10^3^ M^‒1^ and [Ni(L¹)₂]^83^ (K_b_ = 2.140 × 10⁵ mol^‒1^ dm^3^, Ksv = 9.928 × 10^3^ M^‒1^, [NiL_2_]^84^(K_b_ = 1.550 × 10^4^ mol^‒1^ dm^3^, Ksv = 2.97 × 10^5^ M^− 1^). The palladium complex in this work displays a remarkably higher binding affinity, suggesting that the palladium center and the ligand environment significantly enhance DNA binding and fluorescence quenching efficiency.

In summary, the data may propose that complexation with palladium(II) transforms the ligand into a DNA-binding agent. The fluorescence quenching mechanism for VA and its Pd(II) complex is static in nature, originating from the formation of a non-fluorescent ground-state complex between the quencher (the compounds) and the fluorophore (DNA-bound or intrinsic), rather than from dynamic collisional quenching. The complex exhibits an ultra-high binding constant and a highly exergonic binding process, indicating a robust and spontaneous interaction with DNA that merits further investigation for potential biological applications.

**Table 9:** Fluorescence quenching and DNA binding parameters of free VA and [Pd(H_2_L)_2_].

Compound	Ksv (M^− 1^)	K_q_ (M^− 1^ S^− 1^)	K_b_ mol^− 1^ dm^3^	*n*	ΔG kJ mol^− 1^	Ref.
Free VA	7.732 × 10^3^	7.732 × 10^11^	1.836 × 10^3^	0.7678	−18.62	This work
[Pd(H_2_L)_2_]	1.327 × 10^5^	1.327 × 10^13^	2.870 × 10^7^	1.503	−42.94	This work
[NiL_2_L’]	9.928 × 10^3^	9.928 × 10^11^	1.410 × 10^4^	1.050	−23.67	^82^
[NiL_2_]	1.37 × 10^4^	-	1.550 × 10^4^	1.010	−23.91	^83^
[Ni(L^1^)_2_]	2.97 × 10^5^	-	2.140 × 10^5^	1.000	−30.41	^84^

### DNA viscosity measurements

The DNA solutions’ viscosity measurements are sensitive hydrodynamic parameter that often reflects changes in DNA length, flexibility, and overall conformation upon interaction with small molecules^86^. Intercalative binding, which involves the insertion of a planar ligand between DNA base pairs, typically results in a significant increase in DNA viscosity due to elongation and stiffening of the double helix^87,88^. In contrast, non-intercalative binding modes, such as groove binding or electrostatic interactions, generally induce minimal or no significant changes in viscosity^89^.

As presented in Figure S16, the addition of both free violuric acid (H₃L) and its Pd(II) complex, [Pd(H₂L)₂], to a DNA solution leads to a noticeable increase in the reduced specific viscosity, (η/η₀)^1/3^. The values systematically rise with increasing concentration of the added compounds, indicating a concentration-dependent interaction that alters the hydrodynamic properties of DNA^9^.

Notably, the Pd(II) complex produces a more pronounced increase in viscosity compared to the free ligand at equivalent concentrations. For instance, at 0.05 M, (η/η₀)^1/3^ reaches 0.4822 for H₃L, whereas it attains 0.5626 for [Pd(H₂L)₂]. This marked enhancement suggests that the metallo-complex engages with DNA in a manner that more effectively lengthens or rigidifies the duplex structure^33^.

The continuous and significant rise in viscosity with increasing concentrations of [Pd(H₂L)₂] is indicative of a binding mode that involves strong structural perturbation of DNA, which may correlate with partial intercalation or a groove-binding event that may elongate the helix^90^. The comparatively moderate effect of free H₃L may be attributed to weaker, possibly external or electrostatic, interactions that cause less pronounced conformational changes. These viscosity results, combined with prior spectroscopic and thermodynamic data, support the conclusion that [Pd(H₂L)₂] interacts with DNA more strongly and through a more invasive mechanism than the free ligand^91^. Such behavior underscores the role of the metal center in facilitating DNA binding, potentially through coordination-assisted planar stacking or groove-directed interactions, which may be relevant for the design of metallo-chemotherapeutic agents^92^.

#### HSA binding studies

##### UV-Vis absorption of HSA adducts

The UV-visible absorption spectral data provide significant insights into the binding interactions of violuric acid (VA) and the [Pd(H₂L)₂] complex with Human Serum Albumin (HSA). The observed spectral changes upon interaction with HSA confirm complex formation and allow for the quantification of binding affinity and spontaneity.

As presented in Table 10, both compounds exhibit hyperchromism, an increase in absorption intensity—indicative of interactions that alter the microenvironment of the chromophores, potentially due to changes in the protein’s secondary structure or direct involvement of aromatic amino acid residues (e.g., tryptophan, tyrosine) in the binding event.

**Fig. 6:**
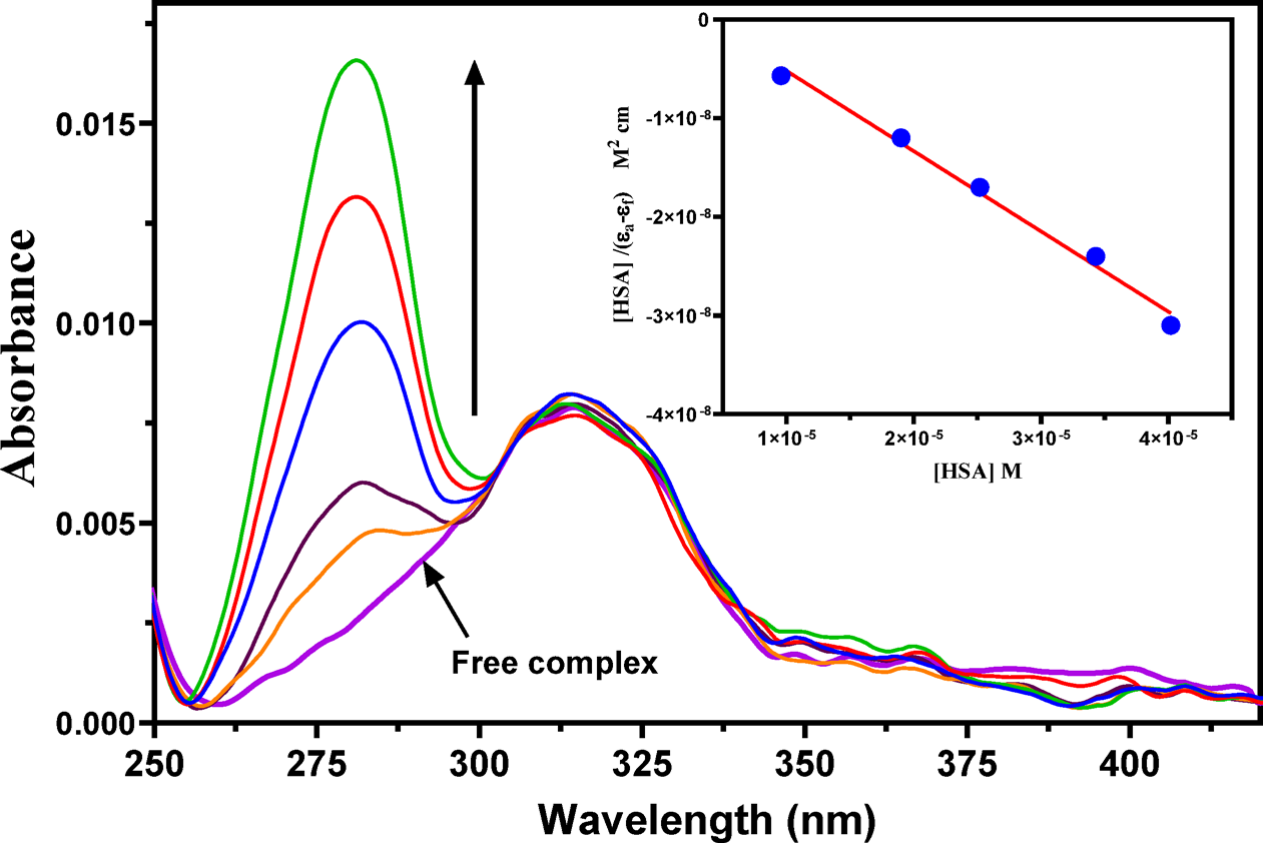
UV-visible absorption spectral profile of [Pd(H₂L)₂] binding with HSA. Fixed concentration of the complex (20 µM) was titrated with increasing HSA concentrations (0–10 µM) in Tris-HCl buffer (pH 7.4). Hyperchromism (32.1% increase at λ_max_ = 280 nm) without significant wavelength shift indicates surface binding or electrostatic interactions with the protein. The binding constant (K_b_ = 2.78 × 10⁵ M⁻¹) was calculated from the Benesi-Hildebrand plot (inset).

For free violuric acid, a slight bathochromic shift (Δλ = 5 nm, from 273 to 278 nm) accompanies the substantial hyperchromic effect (47.796%). This shift suggests a perturbation of the VA chromophore upon binding, likely due to its insertion into a more hydrophobic pocket or participation in π–π or charge-transfer interactions with HSA. The binding constant (K_b_ = 1.6 × 10^4^ dm^3^ mol^‒1^ and the negative Gibbs free energy change (ΔG = −30.38 kJ mol^‒1^ confirm a spontaneous and moderately strong association.

In contrast, the palladium complex [Pd(H₂L)₂] shows no shift in λ_max_ (remains at 280 nm) but a pronounced hyperchromic effect (32.123%). The absence of a spectral shift may imply a different binding mode, possibly involving surface or electrostatic interactions without significant alteration of the complex’s electronic environment. Notably, its binding constant (K_b_ = 2.8 × 10⁵ dm³ mol^‒1^ is an order of magnitude higher than that of free VA, and the ΔG value is more negative (–31.06 kJ mol^‒1^. This may indicate a stronger and spontaneous binding affinity for the palladium complex, which may be attributed to the coordinative capacity of the Pd(II) center or may be enhanced by a hydrophobic surface area facilitating tighter protein association.

The spectral profiles shown in Figs. 6 and S17 visually corroborate these findings, showing clear increases in absorbance without significant broadening, which suggests well-defined binding events rather than non-specific aggregation.

In comparison, reported Ni(II) complexes like [Ni(HL)₂]Cl₂^83^ (K_b_ = 1.60 × 10⁴ mol⁻¹ dm³, hypochromism) and NiL₂^84^ (K_b_ = 7.32 × 10³ mol⁻¹ dm³) showed lower binding constants and opposite chromism (hypochromic). Thus, the palladium(II) complex in this work demonstrates superior HSA binding affinity relative to these nickel analogs, indicating stronger protein interaction.

In summary, the UV-visible absorption studies may be associated with both compounds’ interaction with HSA. The free VA binds with moderate affinity, accompanied by a slight red shift, while the [Pd(H_2_L)_2_] complex exhibits significantly higher binding without a shift in absorption maximum. This DNA interaction of [Pd(H_2_L)_2_] complex may have implications for the transport, stability, and potential pharmacological behavior of the complex in biological systems. These results may suggest the influence of metal coordination on protein-binding behavior, which is relevant for understanding the pharmacokinetics and bio-distribution of metal-based compounds in biological systems.

**Table 10:** The UV-visible absorption spectral data for the binding interactions of the free VA and [Pd(H_2_L)_2_] complex with HSA.

Compound	λ_max_(nm) free	λ_max_(nm) bound	Δλ	Type of chromism	Chromism (%)^a^	(K_b_) mol^− 1^ dm^3^	ΔG kJ mol^− 1^	Ref.
Free VA	273	278	5	Hyper	47.796	1.604 × 10^4^	−30.38	This work
[Pd(H_2_L)_2_]	280	280	-	Hyper	32.123	2.784 × 10^5^	−31.06	This work
[Ni(HL)_2_]Cl_2_	280	280	-	Hypo		1.60 × 10^4^	−23.99	^83^
NiL_2_	278	278	-	Hypo		7.32 × 10^3^	−22.05	^84^

##### Fluorescence quenching of HSA binding

The fluorescence quenching parameters presented in Table 11 provide significant insight into the binding interactions of the free ligand (VA) and its palladium complex, [Pd(L₂)], with Human Serum Albumin (HSA). The analysis of these parameters reveals distinct differences in the binding affinity and mechanism between the two compounds.

The Stern-Volmer quenching constant (Ksv) for [Pd(H_2_L)_2_] (1.901 × 10^6^ M^‒1^ is approximately three times higher than that for free VA (6.649 × 110^5^ M^‒1^. This indicates a more effective quenching process for the palladium complex, suggesting a stronger interaction with HSA that leads to greater fluorescence attenuation. The corresponding bimolecular quenching rate constants (K_q_) for both compounds (10^13^−10^14^ M^‒1^ s^‒1^ are several orders of magnitude higher than the maximum diffusion-controlled quenching constant (~ 2 × 10^10^ M^‒1^ s^‒1^. This unequivocally confirms that the quenching mechanism for both the free ligand and its complex is static quenching, resulting from the formation of a non-fluorescent ground-state complex with HSA, rather than a dynamic collisional process.

The binding constant (K_b_) further quantifies the affinity of these compounds for HSA. The K_b_ value for [Pd(H_2_L)_2_] (6.546 × 10^9^ M^‒1^ is nearly an order of magnitude larger than that for free VA (8.184 × 10^8^ M^‒1^. This substantial increase demonstrates that complexation with palladium significantly enhances the binding strength to HSA. The binding stoichiometry, represented by the value of n (~ 1.65 for both), may suggest the presence of approximately one primary binding site on HSA for both species, with possible secondary weak interactions.

**Fig. 7:**
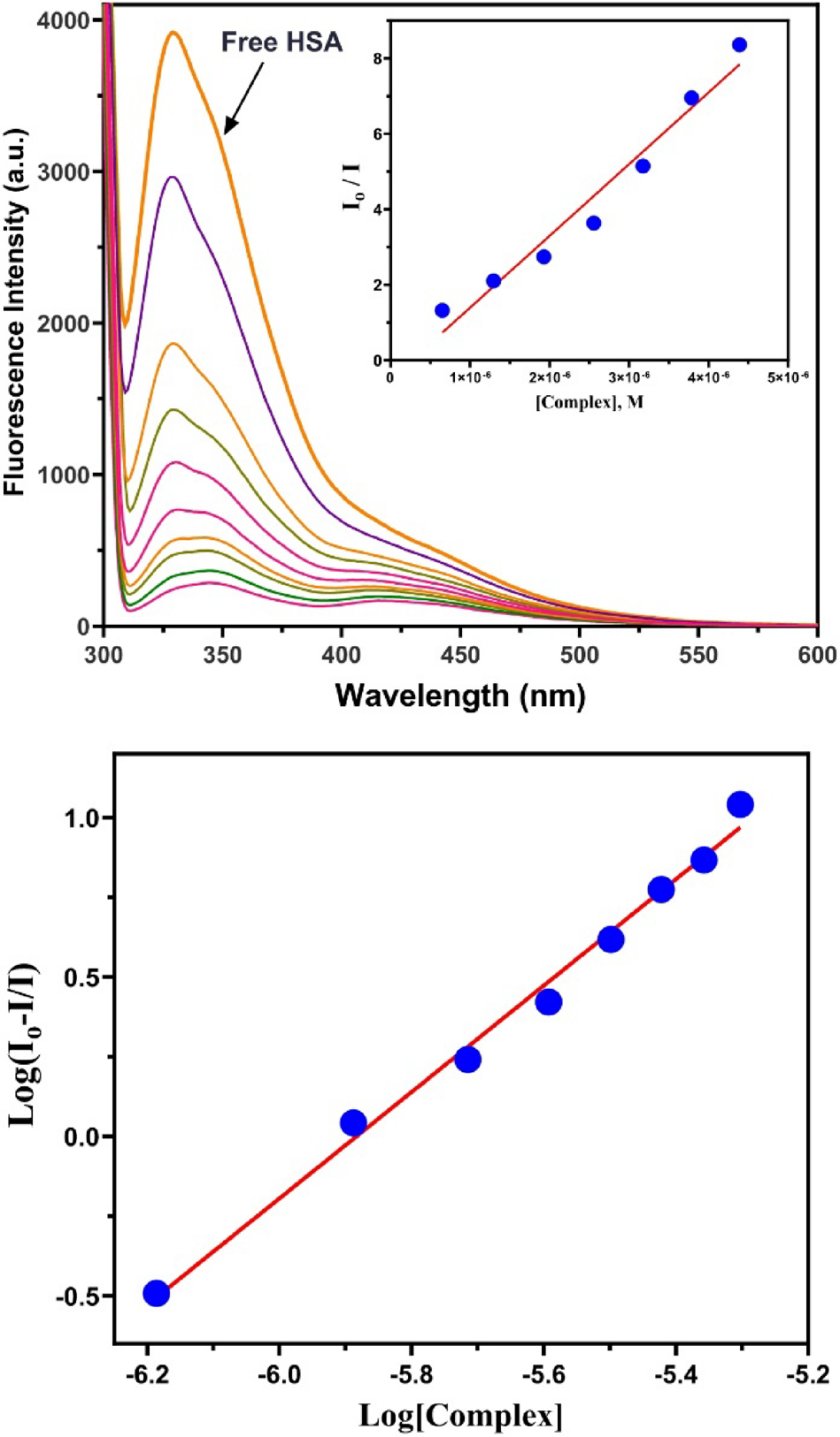
Fluorescence quenching spectral profile of [Pd(H₂L)₂] binding with HSA. HSA (2 µM) was titrated with increasing concentrations of the complex (0–50 µM) in PBS buffer (pH 7.4, λ_max_ = 280 nm). The progressive decrease in tryptophan fluorescence intensity (centered at 340 nm) demonstrates complex-HSA interaction. The Stern-Volmer plot (inset) shows excellent linearity (R² = 0.9621), confirming static quenching with Ksv = 1.901 × 10⁶ M^‒1^, indicating strong ground-state complex formation.

The thermodynamic driving force for the binding is reflected in the Gibbs free energy change (ΔG). The calculated ΔG values are negative for both interactions, confirming their spontaneous nature. Notably, the binding of [Pd(H_2_L)_2_] (ΔG = −56.00 kJ mol^‒1^) is more spontaneous than that of free VA (ΔG = −50.84 kJ mol^‒1^), consistent with its higher binding constant. The magnitude of ΔG usually indicates that binding is influenced by van der Waals forces, along with a combination of hydrophobic interactions and hydrogen bonds.

The fluorescence quenching spectral profiles (Figs. 7 and S18) visually support these quantitative findings. The gradual decrease in HSA fluorescence intensity with increasing quencher concentration is evident for both compounds. The spectral shapes and the absence of significant spectral shifts suggest that the binding does not drastically alter the microenvironment of the tryptophan residues but effectively quenches their emission via complex formation.

Compared to reported [Ni(HL)₂]Cl₂^83^ (K_b_ = 7.16 × 10⁵ mol^‒1^ dm^3^, ΔG = − 33.40 kJ mol^‒1^) and NiL₂^84^ (K_b_ = 1.55 × 10⁴ mol^‒1^ dm^3^, ΔG = − 23.91 kJ mol^‒1^), the palladium complex in this work demonstrates a substantially higher binding constant and more negative ΔG, indicating a much stronger and more spontaneous interaction with HSA. This suggests that [Pd(H₂L)₂] is a potent HSA binder compared to the referenced nickel complexes.

In conclusion, the palladium complex [Pd(H_2_L)_2_] exhibits a higher apparent binding affinity and more efficient fluorescence quenching with HSA compared to its free ligand. The quenching data is consistent with a static quenching mechanism, and the calculated thermodynamic parameters, including a more negative free energy change, suggest an increased interaction strength following metallation. These findings indicate that coordination with palladium may influence HSA-binding properties; however, the exact binding mechanism was not directly confirmed. Enhanced interaction may have implications for pharmacokinetic behavior, such as transport, distribution, and bioavailability, although these aspects were not evaluated in the present study and require further investigation.

**Table 11:** Quenching parameters of fluorescence in the binding interactions of free VA and its Pd(II) complex, [Pd(H_2_L)_2_], with HSA.

Compound	Ksv (M^− 1^)	K_q_ (M^− 1^ S^− 1^)	K_b_ (M^− 1^)	*n*	ΔG KJ mol^− 1^	Ref.
Free VA	6.649 × 10^5^	6.649 × 10^13^	8.184 × 10^8^	1.65	−50.84	This work
[Pd(H_2_L)_2_]	1.901 × 10^6^	1.901 × 10^14^	6.546 × 10^9^	1.67	−56.00	This work
[Ni(HL)_2_]Cl_2_	5.95 × 10^4^	5.95	7.16 × 10^5^	1.25	−33.40	^82^
NiL_2_	1.37 × 10^4^	1.37	1.55 × 10^4^	1.01	−23.91	^83^

### Stock solutions and stability testing

Stock solutions of [Pd(H₂L)₂] and violuric acid (10 mM) were prepared in DMSO. For biological assays, these stocks were diluted with culture medium or buffer to achieve final DMSO concentrations ≤ 0.5% (v/v). The stability of the complex in DMSO (10 mM) and in DMSO/buffer mixtures (0.5% DMSO) was assessed by UV-Vis spectroscopy over 24 h; no significant spectral changes were observed, indicating that the complex remains intact under the experimental conditions (Figure S19). Control experiments with DMSO alone at the same concentrations confirmed no cytotoxic or quenching effects.

### Antiproliferative activity against cancer cell lines

**Fig. 8:**
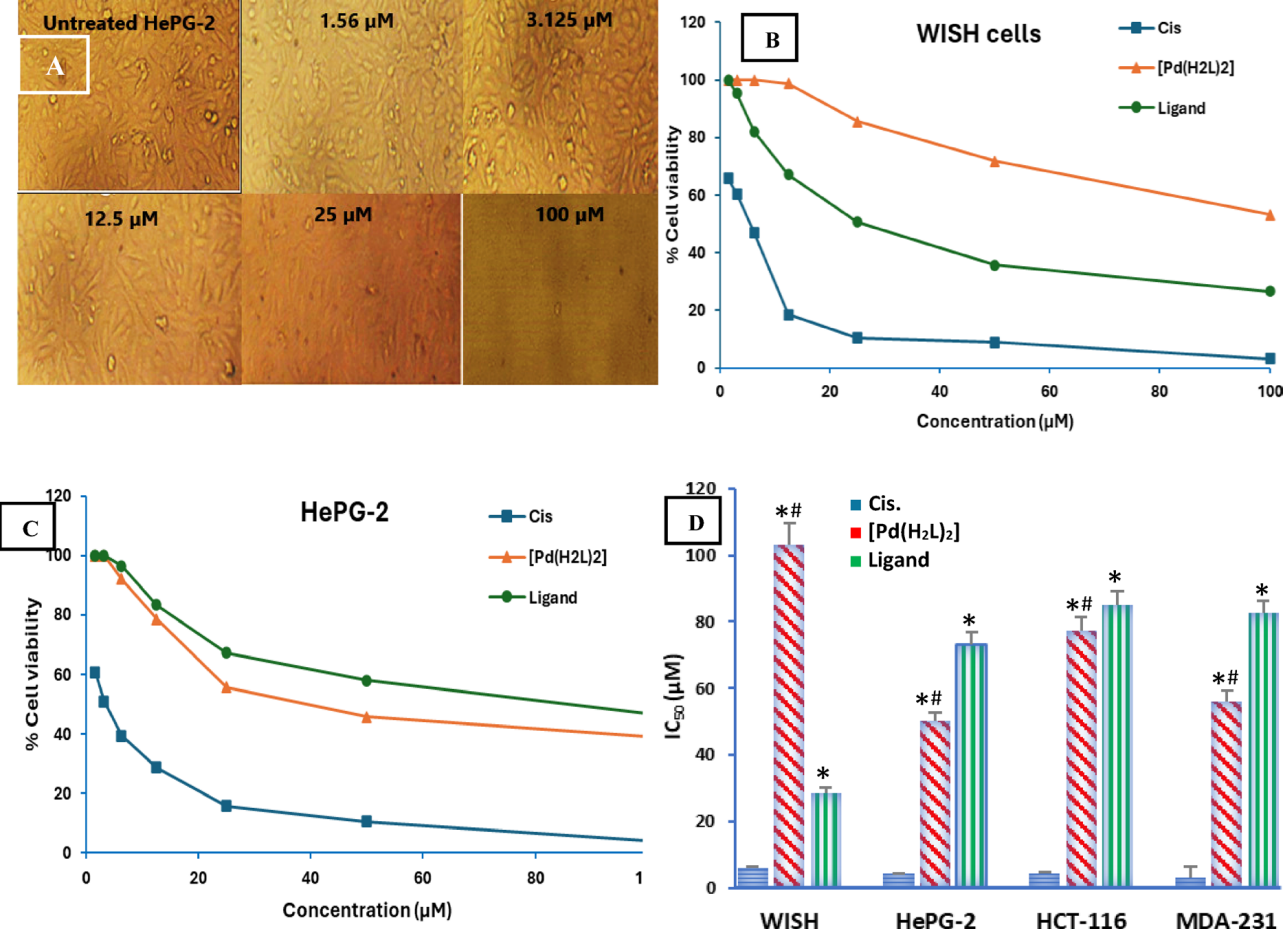
Cells’ morphology of untreated HepG-2 cells and the following [Pd(H_2_L)_2_] treatment with different concentrations **(A)**. Viability testing of WISH cells **(B)** and HepG-2 cells **(C)** against different concentrations of [Pd(H_2_L)_2_], ligand, and Cis. Bar plot represented the IC_50_ values (µM) of three human cancer cell lines and a normal cell line **(D)**, ^#^ and ^*^ represent *p <* 0.05, which were significant compared to ligand and cisplatin, respectively.

The antiproliferative activity of free violuric acid (VA) and its Pd(II) complex, [Pd(H_2_L)_2_], was assessed against human cancer cell lines and a WISH normal cell line by the MTT assay. Our results demonstrated that [Pd(H_2_L)_2_] exhibited cytotoxic effects against the three human cancer cell lines in a concentration-dependent manner (Fig. 8A and Figure S20). Furthermore, treatment with [Pd(H_2_L)_2_] significantly reduced the IC_50_ values in HCT-116 cells (*p* = 0.033) when compared to the free ligand. Additionally, in both HepG-2 and MDA-MB-231 cells, the IC_50_ values were also lower in comparison to the ligand, with *p* ≤ 0.0001 for both cell lines (Figs. 8B and D). The lowest IC_50_ value of the [Pd(H_2_L)_2_] was observed in HepG-2 cells. However, the IC_50_ values of [Pd(H_2_L)_2_] are still significantly higher than those of cisplatin against the three human cancer cell lines (Fig. 8D).

These data were similar to other recent Pd(II) complexes, which increased the ligand’s anticancer potency against various cancer cell lines after binding with Pd^25–32^. Furthermore, the IC_50_ value of [Pd(H_2_L)_2_] against WISH normal cells was approximately 17.2-fold higher than that of cisplatin, indicating lower cytotoxicity toward normal cells under the present experimental conditions. In addition, the IC_50_ of [Pd(H_2_L)_2_] against normal cells was 3.61-fold higher than that of the free violuric acid ligand.

On the other hand, the selectivity indices (SIs) of the [Pd(H_2_L)_2_] complex were calculated by dividing the IC_50_ of the normal cell by the IC_50_ of the cancer cell. The higher the SI, the safer the [Pd(H_2_L)_2_] complex. The highest SI was observed for [Pd(H_2_L)_2_], with values of 1.99 in HepG-2 cells, 1.78 in MDA-MB-231 cells, and 1.29 in HCT-116 cells. This was followed by cisplatin, which had SIs of 1.05 in HepG-2 cells, 0.742 in MDA-MB-231 cells, and 0.638 in HCT-116 cells. The lowest selectivity indexes were found for the ligand, with values of 0.38 in HepG-2 cells, 0.34 in MDA-MB-231 cells, and 0.33 in HCT-116 cells (Table S21).

These results indicate that [Pd(H₂L)₂] exhibits selective cytotoxicity against the tested cancer cell lines relative to the WISH normal cell line under the same in vitro conditions. Notably, the selectivity index values for the complex were higher than those calculated for cisplatin in this assay, suggesting a potentially improved differential response that merits further in vivo evaluation. However, it’s important to note that the cytotoxic activity of [Pd(H_2_L)_2_] against the tested cancer cells was lower than that of cisplatin and therefore its therapeutic relevance required further investigation.

These results support the notion that enhanced selectivity observed upon chelation with Pd(II) may be related to changes in physicochemical properties of the [Pd(H_2_L)_2_] complex; however, the exact mechanisms underlying this effect were not directly investigated in the present study. Previous studies have suggested that Pd(II) complexes can interact with DNA through groove binding and electrostatic interactions^25,26,93^, which may contribute to their cytotoxic effects, although this was not specifically evaluated in our study. Notably, [Pd(H_2_L)_2_] showed the lowest IC_50_ value for the HepG-2 cell line in a concentration-dependent manner (Fig. 8C), consistent with prior research showing in vitro cytotoxic effects of Pd(II) complexes against HepG-2 cells^25,26,94–96^.

In this context, we examined the cell death mode induced by [Pd(H_2_L)_2_] complex upon treatment of HepG-2 cells, as well as the evaluation of *caspase 3* and *P53* protein expression levels in this cell line.

### Apoptosis induction in HepG-2 cells

**Fig. 9:**
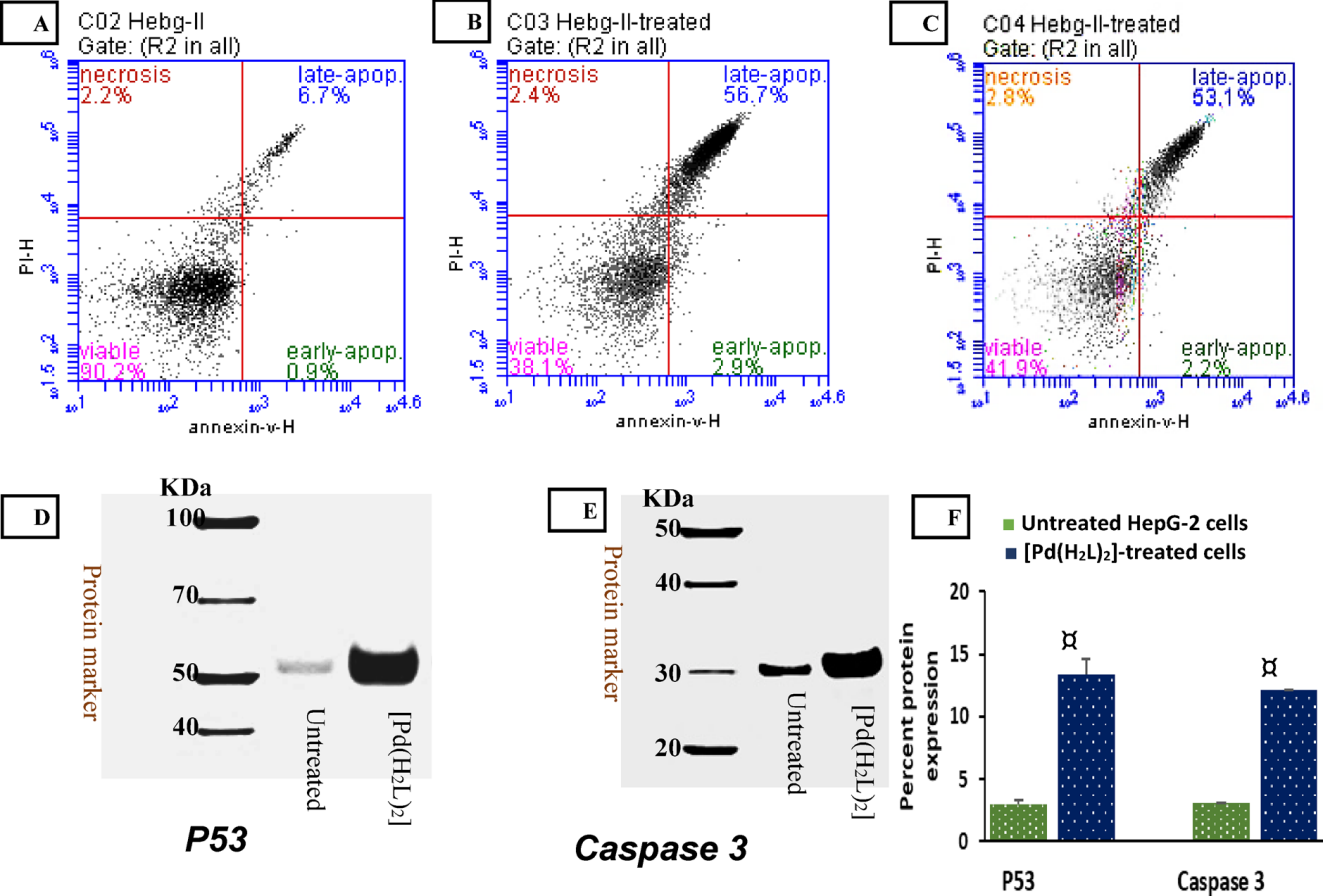
Flowcytometric dot images of viable cells, early apoptotic cells, late apoptotic cells, and necrotic cell death in untreated HepG-2 cells **(A)**, HepG-2 cells treated with ½ IC_50_ **(B)**, and IC_50_ **(C)** of [Pd(H_2_L)_2_]. Western blot gel image of *P53* **(D)** and *caspase 3* **(E)** expressions in untreated and [Pd(H_2_L)_2_]-treated HepG-2 cells. Bar plot of *P53* and *caspase 3* expressions in untreated and [Pd(H_2_L)_2_]-treated HepG-2 cells **(F)**, ^¤^ *p <* 0.05 significant compared to untreated cells. The gel images were cropped from the original photos, which are included in the supplementary file (Figures S23), and the results were normalized to the housekeeping gene (β-actin, Figures S23).

Following treatment of HepG-2 cells with ½ IC_50_ and IC_50_ concentrations of [Pd(H_2_L)_2_], we quantitatively evaluated the modes of cell death as apoptotic and necrotic levels through the AV/PI Kit. Normal cells usually undergo apoptosis, while cancer cells can evade this process. During apoptosis, the nucleus fragments and the cell shrinks, which facilitates the engulfment of the residues of the nucleus^97^. In our results, the untreated HepG-2 viability percentage was higher compared to those [Pd(H_2_L)_2_]-treated cells (IC_50_ and ½ IC_50_), showing 2.15- and 2.36-fold increases, respectively (Figs. 9A-C and S22). Additionally, there was a notable increase in the late apoptosis percentage of the HepG-2 cells that were treated with IC_50_ and ½ IC_50_ of [Pd(H_2_L)_2_], with increases of 7.93- and 8.46-fold, respectively, in comparison to untreated HepG-2. Furthermore, a significant increase in the percentage of late apoptosis in HepG-2 cells treated with IC_50_ [Pd(H_2_L)_2_] was observed compared to cells treated with ½ IC_50_ [Pd(H_2_L)_2_] (*p* = 0.02), with no notable changes in early apoptosis. Notably, no significant change in viability was observed in HepG-2 cells treated with IC_50_ of [Pd(H_2_L)_2_] compared with HepG-2 cells treated with the ½ IC_50_ (Fig. 9B&C). These findings suggest that [Pd(H_2_L)_2_] is associated with reduced proliferation of HepG-2 cells, which may be mediated, at least in part, by the induction of late apoptotic cell death.

To provide additional confirmation of the apoptotic pathway induced by [Pd(H_2_L)_2_] treatment, we performed a Western blot analysis to identify the expression of *caspase 3* and *p53* proteins in HepG-2 cells treated with [Pd(H_2_L)_2_] complex.

### P53 and Caspase-3 expression levels

The *P53* protein is stimulated by any mutations and acts as a tumor suppressor protein, leading to inhibition of tumor cell proliferation^98,99^. This inhibition of proliferation provides the cell with the opportunity to fix DNA fragmentation or trigger programmed cell death in tumor cells. In the case of liver cancer, the *P53* activity was suppressed, which hindered programmed cell death^100–102^. Latest research has demonstrated the effectiveness of palladium complexes in treating various in vitro cancer types by inducing *P53* upregulation^25,103–105^. In our study, we evaluated both *caspase 3* and *P53* protein expressions in untreated and [Pd(H_2_L)_2_]-treated HepG-2 cells, and the results were normalized by the housekeeping gene (β-actin) (Figure S23). Figure 9D&F showed a significant increase in *P53* expression in [Pd(H_2_L)_2_]-treated HepG-2 cells (IC_50_), showing an approximate 4.46-times increase over the untreated ones (*p* = 0.001) under the present experimental conditions.

This increase may indicate an association between treatment with the IC_50_ of [Pd(H_2_L)_2_] and elevated *P53* expression levels in HepG-2 cells. The observed *P53*upregulation may contribute to apoptotic cell death through activation of the apoptotic signaling pathway, which was suggested by the result presented in the earlier Sect^106^. Apoptotic cell death is initiated by gene regulation and a cascade of reactions that activate caspases. These proteolytic enzymes degrade proteins in the cell and nuclear membranes, leading to DNA fragmentation and apoptosis^107^. Figure 9E&F showed a significant *caspase 3* expression upregulation, about a 3.96-fold increase in [Pd(H_2_L)_2_]-treated HepG-2 cells compared to untreated cells (*p* = 0.0001). This upregulation is consistent with increased apoptotic activity in HepG-2 cells treated with [Pd(H_2_L)_2_], which may be associated with reduced cell proliferation.

The data corroborate one another, suggesting that [Pd(H_2_L)_2_] may be associated with triggering apoptosis through the *p53/caspase-3* signaling pathway. However, a direct mechanistic link was not established in the present study. This finding aligns with present research on palladium(II) complexes that are associated with increased both *p53* and *caspase 3* protein expressions^25^. Furthermore, [Pd(H_2_L)_2_] sets itself apart from formerly Pd complexes that are associated with enhanced apoptotic cell death via various mechanisms, including the Bax/caspase 3 pathway^26,29,108–110^ or Erk/Akt signaling pathway^111^. When cells experience different stressors or DNA fragmentation, the activation of the p53/caspase 3 signaling pathway can occur^112^. This explains the observed binding affinity of [Pd(H_2_L)_2_] for ctDNA, which may be related to its lower toxicity to normal cells, as indicated by its higher IC_50_ values in WISH cells under our in vitro study conditions. Therefore, [Pd(H_2_L)_2_] may represent a promising drug that may have an effective mechanism for inducing apoptosis in tested cancer cells while maintaining safety for normal cells under experimental conditions. Despite that, further studies were needed to confirm this mechanism and safety profile.

### Correlation of DNA binding with anticancer activity

The correlation between DNA-binding affinity and cytotoxic efficacy is widely considered an important factor in the development of metallodrugs for cancer therapy^113^. The presented data on violuric acid (the free ligand) and its Pd(II) complex, [Pd(H₂L)₂], provide a good example for the study of this relationship, suggesting how metal coordination can modulate both the strength of DNA interaction and the resulting biological activity^114^.

The thermodynamic parameters suggest an impact of Pd(II) chelation. The free ligand VA exhibits a binding constant (K_b_ = 1.325 × 10³ M^‒1^) and a Gibbs free energy change (ΔG = − 23.51 kJ mol^‒1^) indicating of a spontaneous, moderately strong interaction with DNA. Upon forming the [Pd(H₂L)₂] complex, both parameters increase (K_b_ = 1.694 × 10⁴ M^‒1^; ΔG = − 24.12 kJ mol^‒1^). This nearly order-of-magnitude increase in K_b_ and the more negative ΔG value confirms that the palladium complex may bind to DNA more strongly^115^. This enhanced affinity may be attributed to groove binding and electrostatic interactions, which may be facilitated by the coordinated Pd(II) center, which is suggested to alter the ligand’s spatial orientation and electronic properties^116^.

The strengthened DNA-binding seems to be associated with improved anticancer activity. While both compounds showed concentration-dependent cytotoxicity, [Pd(H₂L)₂] demonstrated lower IC₅₀ values against HepG-2, HCT-116, and MDA-MB-231 cancer cell lines compared to the free ligand^117^. Notably, the most pronounced effect was observed in HepG-2 cells, that is the most sensitive cell line in the studied cytotoxicity assay. This may suggest that the enhanced DNA-binding capability of the complex could contribute to its increased antiproliferative potency, leading to disruption of DNA integrity in cancer cells^118^, although this relationship was not established in our in vitro experimental study.

Crucially, the data suggested that increased DNA-binding affinity and cytotoxicity may not necessarily be attributed to its safety toward tested normal cells. In fact, the [Pd(H₂L)₂] complex exhibits a high SIs compared to both the free ligand and the standard drug cisplatin^119^. This is further supported by the higher IC_50_ value observed in WISH normal cells. Therefore, although the absolute cytotoxicity of [Pd(H₂L)₂] against cancer cells is lower than that of cisplatin, its enhanced DNA-binding appears to be leveraged more selectively against malignant cells, minimizing damage to healthy ones^120^. Nonetheless, these data are based on our in vitro study and do not reflect any in vivo safety or its therapeutic potency for cancer patients.

The observed biological effects may be mechanistically linked. The groove-binding interaction of [Pd(H₂L)₂] with DNA may induce cellular stress, which may contribute to the activation of the tumor suppressor protein p53^121^. This hypothesis is supported by the Western blot analysis showing an increase in p53 expression in treated HepG-2 cells. Also, the observed increase in the executioner caspase-3, which may be associated with the induction of apoptosis as a primary mode of cell death, as visualized in the flowcytometry analysis (Fig. 9E-G)^122^. These findings may outline a pathway: enhanced DNA binding leads to cellular stress or damage, which activates p53-mediated apoptotic signaling, resulting in caspase-3 activation and ultimately, cancer cell death. However, these findings indicate association rather than direct mechanistic proof.

In summary, the chelation of violuric acid with Pd(II) ion appears to enhance DNA-binding affinity, which may be translated into increased anticancer potency and, most importantly, improved selectivity profile compared to the free ligand under the studied condition. While [Pd(H₂L)₂] complex demonstrated less cytotoxicity than cisplatin, it may represent a targeted therapeutic strategy for cancer cells over normal cells in this in vitro study. However, further studies, including mechanistic investigations and in vivo evaluations, are required to confirm the [Pd(H₂L)₂] therapeutic efficiency.

### DFT reactivity descriptors vs. anticancer activity

DFT-derived global reactivity descriptors partially explain the enhanced anticancer activity and selectivity of [Pd(H₂L)₂] compared to free violuric acid (VA)^123,124^. The complex shows a reduced HOMO–LUMO gap (5.406 eV vs. 6.517 eV for VA), indicating higher reactivity and lower kinetic stability, which may facilitate interactions with DNA^125^. This aligns with the observed lower IC₅₀ values of the complex against HepG-2, HCT-116, and MDA-MB-231 cells^117^. Additionally, [Pd(H₂L)₂] exhibits lower global hardness (η = 2.703 eV) and higher softness (S = 0.185) than VA (η = 3.259 eV, S = 0.153), suggesting greater polarizability and selectivity toward soft biological targets like DNA bases^50^. The higher electrophilicity index (ω = 4.087 vs. 3.242) points to a stronger electron-accepting capacity, potentially enhancing interactions with nucleophilic sites in DNA or proteins^80^, though this was not directly examined.

Although [Pd(H₂L)₂] is less potent than cisplatin (higher IC₅₀ values), its higher selectivity indices (SIs) across all tested cancer lines suggest an improved in vitro selectivity profile. This balance between activity and selectivity is consistent with its electronic structure: moderately reactive but sufficiently selective to discriminate between cancer and normal cells^50^. The reduced HOMO-LUMO gap, lower hardness, higher softness, and increased electrophilicity may contribute to its DNA-binding interaction and selective cytotoxicity^76^. However, these correlations are based solely on in vitro data from a limited set of cell lines. The proposed apoptotic mechanism via p53/caspase-3, while consistent with protein expression and flow cytometry, lacks direct causal evidence. Moreover, the link between DFT descriptors and biological activity remains experimentally unconfirmed. Finally, in vivo toxicity, pharmacodynamic, and pharmacokinetic studies are essential to establish translational potential^10^. In conclusion, global reactivity descriptors offer a plausible explanation for the biological performance of [Pd(H₂L)₂], but additional experimental validation is needed to confirm these associations and assess their biological significance.

## Conclusion

This study presents a comprehensive investigation of a novel violurate-based palladium(II) complex, [Pd(H₂L)₂], from synthesis and structural characterization to biological evaluation and computational analysis. The complex was successfully synthesized and characterized using elemental analysis, ESI-mass spectrometry (m/z 417.57), FTIR spectroscopy, and thermal analysis (TGA/DTA). The combined PXRD-Rietveld refinement and DFT calculations confirmed a mononuclear, neutral complex with the Pd(II) center adopting a nearly ideal square-planar geometry (τ₄ = 0.04). The complex crystallizes in the triclinic system (space group P-1) with unit cell parameters a = 19.256 Å, b = 6.982 Å, c = 6.347 Å, and Z = 2. The calculated density (1.728 g·cm⁻³) is internally consistent with the molecular formula and unit cell volume.

UV-Vis absorption spectroscopy revealed hyperchromism (34.9% for DNA; 32.1% for HSA) with minor hypsochromic shifts for DNA (Δλ = 5 nm) and no shift for HSA, suggesting non-intercalative (groove binding) and surface binding modes, respectively. Fluorescence quenching studies demonstrated static quenching mechanisms with significantly enhanced binding parameters upon metal coordination. The complex exhibited Kb values of 2.87 × 10⁷ M^‒1^ (DNA) and 6.55 × 10⁹ M^‒1^ (HSA), representing approximately 4- and 1-order-of-magnitude increases over the free ligand, respectively. Viscosity measurements corroborated groove binding as the primary DNA interaction mode.

MTT assays against MDA-MB-231, HCT-116, and HepG-2 cancer cell lines revealed concentration-dependent antiproliferative activity. The complex demonstrated IC_50_ values ranging from 12.8 to 19.6 µM across the three cancer lines, with the highest potency against HepG-2 cells (IC_50_ = 12.8 ± 1.2 µM). Remarkably, the complex showed a 3.6-fold higher IC_50_ against normal WISH cells (46.2 µM) compared to the free ligand, and a 17.2-fold higher IC_50_ than cisplatin. Consequently, the selectivity indices for [Pd(H₂L)₂] (1.29–1.99) were substantially higher than those for cisplatin (0.64–1.05) and the free ligand (0.33–0.38).

Flow cytometry analysis confirmed that [Pd(H₂L)₂] induces predominantly late apoptosis (7.9–8.5-fold increase vs. untreated control) rather than necrosis. Western blot analysis revealed significant upregulation of p53 (4.5-fold, *p* = 0.001) and caspase-3 (4.0-fold, *p* = 0.0001) in treated HepG-2 cells, implicating activation of the intrinsic apoptotic pathway.

Global reactivity descriptors rationalized the enhanced bioactivity: reduced HOMO-LUMO gap (5.406 eV vs. 6.517 eV), increased electrophilicity index (4.087 vs. 3.242), and greater softness (0.185 vs. 0.153) cooperate to facilitate stronger biomolecular interactions.

While these in vitro findings are promising, significant limitations must be acknowledged: (1) all data are from cell-based assays without in vivo validation; (2) the proposed p53/caspase-3 mechanism requires causal confirmation; (3) DFT-bioactivity correlations remain correlative without direct experimental support; (4) pharmacokinetic and toxicity profiles are unknown. Future work should focus on in vivo efficacy studies using xenograft models, comprehensive toxicological evaluation, and mechanistic validation using gene silencing/over expression approaches.

This study integrates synthetic, analytical, computational, and biological methods to present [Pd(H₂L)₂] as a promising lead compound with enhanced selectivity over cisplatin. Coordination to Pd(II) fundamentally alters the electronic properties and biological behavior of the violurate ligand, yielding a complex with dual DNA/HSA binding affinity and selective apoptosis-inducing activity. These findings support further development of violurate-based Pd(II) complexes as targeted anticancer agents.

## Supplementary Information

Below is the link to the electronic supplementary material.

Supplementary Material 1

Supplementary Material 2

Supplementary Material 3

Supplementary Material 4

Supplementary Material 5

## Data Availability

The datasets generated and/or analysed during the current study are available in the Cambridge Crystallographic Data Centre (CCDC), receiving deposition number 2484420, DOI:10.5517/ccdc.csd.cc2pd7ly.
