# Supplementary material for: Synthesis, structural characterization, and dual DNA/HSA binding of novel Palladium(II) violurate complex with selective p53/Caspase-3-mediated anticancer activity
Source: Sci Rep. 2026 Jun 27;16:19632. doi: 10.1038/s41598-026-58248-w (PMC13310190; doi:10.1038/s41598-026-58248-w)

checkCIF/PLATON report

Structure factors have been supplied for datablock(s) I

THIS REPORT IS FOR GUIDANCE ONLY. IF USED AS PART OF A REVIEW PROCEDURE FOR PUBLICATION, IT SHOULD NOT REPLACE THE EXPERTISE OF AN EXPERIENCED CRYSTALLOGRAPHIC REFEREE.

No syntax errors found. CIF dictionary Interpreting this report

Datablock: I

```
Bond precision:   C-C = 0.0011 A          Wavelength=1.54056

Cell:             a=19.256(8)        b=6.982(5)        c=6.347(4)
                  alpha=108.62(4)    beta=94.44(4)     gamma=84.77(3)
Temperature:      0 K

                  Calculated                   Reported
Volume            804.2(9)                     804.3(9)
Space group       P -1                         P -1
Hall group        -P 1                         -P 1
Moiety formula    C8 H4 N6 O8 Pd               C8 H4 N6 O8 Pd
Sum formula       C8 H4 N6 O8 Pd               C8 H4 N6 O8 Pd
Mr                418.57                       418.57
Dx,g cm-3         1.729                        1.728
Z                 2                            2
Mu (mm-1)         9.797                        9.767
F000              408.0                        0.0
F000'             409.70
h,k,lmax          9,3,3                        8,3,2
Nref              180                          140
Tmin,Tmax
Tmin'

Correction method= Not given
```

```
Data completeness= 0.778            Theta(max)= 21.500

R(reflections)=                                        wR2(reflections)=
S =                          Npar=
```

The following ALERTS were generated. Each ALERT has the format
test-name_ALERT_alert-type_alert-level.
Click on the hyperlinks for more details of the test.

Alert level A

```
DIFF003_ALERT_1_A  _diffrn_measurement_device_type is missing
            Diffractometer make and type. Replaces _diffrn_measurement_type.
PLAT197_ALERT_1_A Missing _cell_measurement_temperature Datum ....     Please Add
PLAT198_ALERT_1_A Missing _diffrn_ambient_temperature   Datum ....     Please Add
PLAT602_ALERT_2_A Solvent Accessible VOID(S) in the Unit-Cell ....          ! Check
PLAT902_ALERT_1_A No (Interpretable) Reflections Found in FCF ....     Please Check
```

Alert level B

```
REFLL01_ALERT_1_B  The minimum h value cannot exceed the maximum h value
            Minimum h value =    100
            Maximum h value =   -100
REFLL01_ALERT_1_B  The minimum k value cannot exceed the maximum k value
            Minimum k value =    100
            Maximum k value =   -100
REFLL01_ALERT_1_B  The minimum l value cannot exceed the maximum l value
            Minimum l value =    100
            Maximum l value =   -100
PLAT369_ALERT_2_B Long   C(sp2)-C(sp2) Bond  C1        - C2       .       1.57 Ang.
PLAT369_ALERT_2_B Long   C(sp2)-C(sp2) Bond  C5        - C6       .       1.58 Ang.
```

Alert level C

```
REFI015_ALERT_1_C  _refine_ls_shift/su_max is missing
            Maximum shift/s.u. ratio after final refinement cycle.
            The following tests will not be performed
            SHFSU_01
PLAT155_ALERT_4_C The Triclinic Unit-Cell is NOT Reduced .........     Please Do !
              INP:  19.256   6.982   6.347  108.62   94.44   84.77     804.200
              RED:   6.347   6.982  19.256   84.77   85.56   71.38     804.200
PLAT242_ALERT_2_C Low   MainResAtom  Ueq as Compared to Neighbours        Pd1 Check
PLAT353_ALERT_3_C Long   N-H (N0.87,N1.01A)  N3        - H1       .       1.07 Ang.
PLAT353_ALERT_3_C Long   N-H (N0.87,N1.01A)  N4        - H2       .       1.07 Ang.
PLAT353_ALERT_3_C Long   N-H (N0.87,N1.01A)  N5        - H3       .       1.04 Ang.
PLAT420_ALERT_2_C D-H Bond Without Acceptor  N3        --H1       .     Please Check
PLAT420_ALERT_2_C D-H Bond Without Acceptor  N4        --H2       .     Please Check
PLAT420_ALERT_2_C D-H Bond Without Acceptor  N5        --H3       .     Please Check
PLAT741_ALERT_1_C Bond    Calc  1.3335(10), Rep     1.33371 ......    Missing s.u.
              N1       -O1               1_555   1_555 ........     #    1 Check
PLAT741_ALERT_1_C Bond    Calc  1.3551(10), Rep     1.35544 ......    Missing s.u.
              N1       -C5               1_555   1_555 ........     #    2 Check
PLAT741_ALERT_1_C Bond    Calc  2.1172(15), Rep     2.11737 ......    Missing s.u.
              O1       -PD1              1_555   1_555 ........     #    3 Check
```

```
PLAT741_ALERT_1_C Bond    Calc  1.4094(10), Rep     1.40919 ......    Missing s.u.
             O1       -O8                1_555   1_566 ........     #    4 Check
PLAT741_ALERT_1_C Bond    Calc  2.0649(15), Rep     2.06487 ......    Missing s.u.
             PD1      -O2                1_555   1_555 ........     #    5 Check
PLAT741_ALERT_1_C Bond    Calc  2.1779(16), Rep     2.17863 ......    Missing s.u.
             PD1      -O3                1_555   1_555 ........     #    6 Check
PLAT741_ALERT_1_C Bond    Calc  2.1241(15), Rep     2.12468 ......    Missing s.u.
             PD1      -O4                1_555   1_555 ........     #    7 Check
PLAT741_ALERT_1_C Bond    Calc  1.3410(10), Rep     1.34081 ......    Missing s.u.
             O2       -N2                1_555   1_555 ........     #    8 Check
PLAT741_ALERT_1_C Bond    Calc  1.3858(10), Rep     1.38520 ......    Missing s.u.
             N2       -C1                1_555   1_555 ........     #   10 Check
PLAT741_ALERT_1_C Bond    Calc  1.5704(11), Rep     1.57030 ......    Missing s.u.
             C1       -C2                1_555   1_555 ........     #   11 Check
PLAT741_ALERT_1_C Bond    Calc  1.4910(11), Rep     1.49135 ......    Missing s.u.
             C1       -C4                1_555   1_555 ........     #   12 Check
PLAT741_ALERT_1_C Bond    Calc   1.2392(9), Rep     1.23960 ......    Missing s.u.
             C2       -O6                1_555   1_555 ........     #   13 Check
PLAT741_ALERT_1_C Bond    Calc  1.4712(11), Rep     1.47178 ......    Missing s.u.
             C2       -N4                1_555   1_555 ........     #   14 Check
PLAT741_ALERT_1_C Bond    Calc   1.2696(9), Rep     1.27039 ......    Missing s.u.
             C3       -O5                1_555   1_555 ........     #   15 Check
PLAT741_ALERT_1_C Bond    Calc  1.4087(10), Rep     1.40837 ......    Missing s.u.
             C3       -N4                1_555   1_555 ........     #   16 Check
PLAT741_ALERT_1_C Bond    Calc  1.4818(11), Rep     1.48131 ......    Missing s.u.
             C3       -N5                1_555   1_555 ........     #   17 Check
PLAT741_ALERT_1_C Bond    Calc   1.3154(9), Rep     1.31513 ......    Missing s.u.
             C4       -O3                1_555   1_555 ........     #   18 Check
PLAT741_ALERT_1_C Bond    Calc  1.4392(10), Rep     1.43939 ......    Missing s.u.
             C4       -N5                1_555   1_555 ........     #   19 Check
PLAT741_ALERT_1_C Bond    Calc  1.5781(11), Rep     1.57837 ......    Missing s.u.
             C5       -C6                1_555   1_555 ........     #   20 Check
PLAT741_ALERT_1_C Bond    Calc  1.5198(11), Rep     1.51963 ......    Missing s.u.
             C5       -C8                1_555   1_555 ........     #   21 Check
PLAT741_ALERT_1_C Bond    Calc  1.4394(10), Rep     1.43954 ......    Missing s.u.
             C6       -N3                1_555   1_555 ........     #   22 Check
PLAT741_ALERT_1_C Bond    Calc   1.2675(9), Rep     1.26684 ......    Missing s.u.
             C6       -O7                1_555   1_555 ........     #   23 Check
PLAT741_ALERT_1_C Bond    Calc  1.4331(10), Rep     1.43308 ......    Missing s.u.
             C7       -N3                1_555   1_555 ........     #   24 Check
PLAT741_ALERT_1_C Bond    Calc   1.2407(9), Rep     1.24095 ......    Missing s.u.
             C7       -O8                1_555   1_555 ........     #   25 Check
PLAT741_ALERT_1_C Bond    Calc  1.4843(11), Rep     1.48406 ......    Missing s.u.
             C7       -N6                1_555   1_555 ........     #   26 Check
PLAT741_ALERT_1_C Bond    Calc  1.3297(10), Rep     1.32973 ......    Missing s.u.
             C8       -O4                1_555   1_555 ........     #   27 Check
PLAT741_ALERT_1_C Bond    Calc  1.4038(10), Rep     1.40415 ......    Missing s.u.
             C8       -N6                1_555   1_555 ........     #   28 Check
PLAT741_ALERT_1_C Bond    Calc  1.4094(10), Rep     1.40919 ......    Missing s.u.
             O8       -O1                1_555   1_544 ........     #   30 Check
PLAT742_ALERT_1_C Angle   Calc   124.98(4), Rep      124.98 ......    Missing s.u.
             O1      -N1      -C5          1_555  1_555  1_555     #    1 Check
PLAT742_ALERT_1_C Angle   Calc   128.29(4), Rep      128.28 ......    Missing s.u.
             N1      -O1      -PD1         1_555  1_555  1_555     #    2 Check
PLAT742_ALERT_1_C Angle   Calc    87.24(5), Rep       87.26 ......    Missing s.u.
             O1      -PD1     -O2          1_555  1_555  1_555     #    5 Check
PLAT742_ALERT_1_C Angle   Calc   177.46(1), Rep      177.46 ......    Missing s.u.
```

```
              O1      -PD1     -O3         1_555  1_555  1_555     #    6 Check
PLAT742_ALERT_1_C Angle   Calc    90.07(5), Rep       90.08 ......    Missing s.u.
              O1      -PD1     -O4         1_555  1_555  1_555     #    7 Check
PLAT742_ALERT_1_C Angle   Calc    90.22(5), Rep       90.21 ......    Missing s.u.
              O2      -PD1     -O3         1_555  1_555  1_555     #    8 Check
PLAT742_ALERT_1_C Angle   Calc   177.32(1), Rep      177.33 ......    Missing s.u.
              O2      -PD1     -O4         1_555  1_555  1_555     #    9 Check
PLAT742_ALERT_1_C Angle   Calc    92.47(5), Rep       92.46 ......    Missing s.u.
              O3      -PD1     -O4         1_555  1_555  1_555     #   10 Check
PLAT742_ALERT_1_C Angle   Calc   126.80(4), Rep      126.81 ......    Missing s.u.
              PD1     -O2      -N2         1_555  1_555  1_555     #   11 Check
PLAT742_ALERT_1_C Angle   Calc   127.09(4), Rep      127.11 ......    Missing s.u.
              O2      -N2      -C1         1_555  1_555  1_555     #   14 Check
PLAT742_ALERT_1_C Angle   Calc   115.09(4), Rep      115.11 ......    Missing s.u.
              N2      -C1      -C2         1_555  1_555  1_555     #   15 Check
PLAT742_ALERT_1_C Angle   Calc   126.04(4), Rep      126.05 ......    Missing s.u.
              N2      -C1      -C4         1_555  1_555  1_555     #   16 Check
PLAT742_ALERT_1_C Angle   Calc   118.87(4), Rep      118.84 ......    Missing s.u.
              C2      -C1      -C4         1_555  1_555  1_555     #   17 Check
PLAT742_ALERT_1_C Angle   Calc   124.97(4), Rep      124.97 ......    Missing s.u.
              C1      -C2      -O6         1_555  1_555  1_555     #   18 Check
PLAT742_ALERT_1_C Angle   Calc   116.21(4), Rep      116.23 ......    Missing s.u.
              C1      -C2      -N4         1_555  1_555  1_555     #   19 Check
PLAT742_ALERT_1_C Angle   Calc   118.82(5), Rep      118.80 ......    Missing s.u.
              O6      -C2      -N4         1_555  1_555  1_555     #   20 Check
PLAT742_ALERT_1_C Angle   Calc   124.03(4), Rep      124.00 ......    Missing s.u.
              O5      -C3      -N4         1_555  1_555  1_555     #   21 Check
PLAT742_ALERT_1_C Angle   Calc   123.31(4), Rep      123.29 ......    Missing s.u.
              O5      -C3      -N5         1_555  1_555  1_555     #   22 Check
PLAT742_ALERT_1_C Angle   Calc   112.66(4), Rep      112.70 ......    Missing s.u.
              N4      -C3      -N5         1_555  1_555  1_555     #   23 Check
PLAT742_ALERT_1_C Angle   Calc   126.07(3), Rep      126.06 ......    Missing s.u.
              C1      -C4      -O3         1_555  1_555  1_555     #   24 Check
PLAT742_ALERT_1_C Angle   Calc   115.80(5), Rep      115.82 ......    Missing s.u.
              C1      -C4      -N5         1_555  1_555  1_555     #   25 Check
PLAT742_ALERT_1_C Angle   Calc   118.13(5), Rep      118.12 ......    Missing s.u.
              O3      -C4      -N5         1_555  1_555  1_555     #   26 Check
PLAT742_ALERT_1_C Angle   Calc   113.01(4), Rep      112.98 ......    Missing s.u.
              N1      -C5      -C6         1_555  1_555  1_555     #   27 Check
PLAT742_ALERT_1_C Angle   Calc   125.83(4), Rep      125.81 ......    Missing s.u.
              N1      -C5      -C8         1_555  1_555  1_555     #   28 Check
PLAT742_ALERT_1_C Angle   Calc   121.17(4), Rep      121.20 ......    Missing s.u.
              C6      -C5      -C8         1_555  1_555  1_555     #   29 Check
PLAT742_ALERT_1_C Angle   Calc   114.20(4), Rep      114.14 ......    Missing s.u.
              C5      -C6      -N3         1_555  1_555  1_555     #   30 Check
PLAT742_ALERT_1_C Angle   Calc   127.07(4), Rep      127.10 ......    Missing s.u.
              C5      -C6      -O7         1_555  1_555  1_555     #   31 Check
PLAT742_ALERT_1_C Angle   Calc   118.73(5), Rep      118.76 ......    Missing s.u.
              N3      -C6      -O7         1_555  1_555  1_555     #   32 Check
PLAT742_ALERT_1_C Angle   Calc   123.52(5), Rep      123.50 ......    Missing s.u.
              N3      -C7      -O8         1_555  1_555  1_555     #   33 Check
PLAT742_ALERT_1_C Angle   Calc   115.17(4), Rep      115.17 ......    Missing s.u.
              N3      -C7      -N6         1_555  1_555  1_555     #   34 Check
PLAT742_ALERT_1_C Angle   Calc   121.31(4), Rep      121.34 ......    Missing s.u.
              O8      -C7      -N6         1_555  1_555  1_555     #   35 Check
PLAT742_ALERT_1_C Angle   Calc   128.26(4), Rep      128.31 ......    Missing s.u.
              C5      -C8      -O4         1_555  1_555  1_555     #   36 Check
```

```
PLAT742_ALERT_1_C Angle   Calc   115.11(5), Rep      115.10 ......    Missing s.u.
             C5      -C8      -N6          1_555  1_555  1_555     #   37 Check
PLAT742_ALERT_1_C Angle   Calc   116.63(4), Rep      116.60 ......    Missing s.u.
             O4      -C8      -N6          1_555  1_555  1_555     #   38 Check
PLAT742_ALERT_1_C Angle   Calc   123.78(4), Rep      123.77 ......    Missing s.u.
             PD1     -O3      -C4          1_555  1_555  1_555     #   39 Check
PLAT742_ALERT_1_C Angle   Calc   122.57(4), Rep      122.54 ......    Missing s.u.
             PD1     -O4      -C8          1_555  1_555  1_555     #   40 Check
PLAT742_ALERT_1_C Angle   Calc   126.82(4), Rep      126.86 ......    Missing s.u.
             C6      -N3      -C7          1_555  1_555  1_555     #   41 Check
PLAT742_ALERT_1_C Angle   Calc   127.25(4), Rep      127.22 ......    Missing s.u.
             C2      -N4      -C3          1_555  1_555  1_555     #   45 Check
PLAT742_ALERT_1_C Angle   Calc   129.21(4), Rep      129.19 ......    Missing s.u.
             C3      -N5      -C4          1_555  1_555  1_555     #   48 Check
PLAT742_ALERT_1_C Angle   Calc   127.53(4), Rep      127.53 ......    Missing s.u.
             C7      -N6      -C8          1_555  1_555  1_555     #   51 Check
PLAT743_ALERT_1_C Torsion Calc  -179.90(4), Rep     -179.96 ......    Missing s.u.
             N1  -O1  -PD1 -O2    1_555  1_555  1_555  1_555     #    1 Check
PLAT743_ALERT_1_C Torsion Calc    -0.13(4), Rep        0.03 ......    Missing s.u.
             O2  -N2  -C1  -C4    1_555  1_555  1_555  1_555     #   18 Check
PLAT743_ALERT_1_C Torsion Calc   179.90(4), Rep      180.00 ......    Missing s.u.
             N2  -C1  -C2  -N4    1_555  1_555  1_555  1_555     #   20 Check
PLAT743_ALERT_1_C Torsion Calc     0.14(4), Rep        0.03 ......    Missing s.u.
             N2  -C1  -C4  -O3    1_555  1_555  1_555  1_555     #   21 Check
PLAT743_ALERT_1_C Torsion Calc  -179.91(4), Rep     -179.98 ......    Missing s.u.
             N2  -C1  -C4  -N5    1_555  1_555  1_555  1_555     #   22 Check
PLAT743_ALERT_1_C Torsion Calc     0.11(4), Rep        0.02 ......    Missing s.u.
             C1  -C2  -N4  -C3    1_555  1_555  1_555  1_555     #   23 Check
PLAT743_ALERT_1_C Torsion Calc    -0.06(4), Rep       -0.03 ......    Missing s.u.
             C1  -C4  -O3  -PD1   1_555  1_555  1_555  1_555     #   25 Check
PLAT743_ALERT_1_C Torsion Calc  -179.93(4), Rep      179.97 ......    Missing s.u.
             C4  -C1  -C2  -O6    1_555  1_555  1_555  1_555     #   30 Check
PLAT743_ALERT_1_C Torsion Calc    -0.08(4), Rep       -0.03 ......    Missing s.u.
             C5  -N1  -O1  -PD1   1_555  1_555  1_555  1_555     #   32 Check
PLAT743_ALERT_1_C Torsion Calc   146.31(4), Rep      146.30 ......    Missing s.u.
             C5  -N1  -O1  -O8    1_555  1_555  1_555  1_566     #   33 Check
PLAT743_ALERT_1_C Torsion Calc    -0.06(4), Rep        0.03 ......    Missing s.u.
             C5  -C8  -O4  -PD1   1_555  1_555  1_555  1_555     #   36 Check
PLAT743_ALERT_1_C Torsion Calc  -179.92(4), Rep     -179.97 ......    Missing s.u.
             C6  -C5  -C8  -O4    1_555  1_555  1_555  1_555     #   39 Check
PLAT743_ALERT_1_C Torsion Calc   179.92(4), Rep      180.00 ......    Missing s.u.
             C8  -C5  -C6  -O7    1_555  1_555  1_555  1_555     #   43 Check
PLAT743_ALERT_1_C Torsion Calc   179.92(4), Rep     -180.00 ......    Missing s.u.
             O3  -C4  -N5  -C3    1_555  1_555  1_555  1_555     #   48 Check
PLAT743_ALERT_1_C Torsion Calc   179.92(4), Rep      179.96 ......    Missing s.u.
             O4  -C8  -N6  -C7    1_555  1_555  1_555  1_555     #   53 Check
PLAT743_ALERT_1_C Torsion Calc   -69.27(4), Rep      -69.25 ......    Missing s.u.
             N3  -C7  -O8  -O1    1_555  1_555  1_555  1_544     #   55 Check
PLAT743_ALERT_1_C Torsion Calc   179.93(4), Rep      180.00 ......    Missing s.u.
             O5  -C3  -N5  -C4    1_555  1_555  1_555  1_555     #   60 Check
PLAT743_ALERT_1_C Torsion Calc  -179.93(4), Rep      179.98 ......    Missing s.u.
             O7  -C6  -N3  -C7    1_555  1_555  1_555  1_555     #   64 Check
PLAT743_ALERT_1_C Torsion Calc    23.67(4), Rep       23.66 ......    Missing s.u.
             O8  -O1  -PD1 -O2    1_566  1_555  1_555  1_555     #   66 Check
PLAT743_ALERT_1_C Torsion Calc  -156.38(4), Rep     -156.33 ......    Missing s.u.
             O8  -O1  -PD1 -O4    1_566  1_555  1_555  1_555     #   68 Check
PLAT743_ALERT_1_C Torsion Calc  -179.91(4), Rep     -180.00 ......    Missing s.u.
```

```
             O8  -C7  -N3  -C6    1_555  1_555  1_555  1_555      #   69 Check
PLAT743_ALERT_1_C Torsion Calc   179.94(4), Rep     -179.98 ......    Missing s.u.
             O8  -C7  -N6  -C8    1_555  1_555  1_555  1_555      #   71 Check
PLAT743_ALERT_1_C Torsion Calc     0.12(4), Rep        0.00 ......    Missing s.u.
             N4  -C3  -N5  -C4    1_555  1_555  1_555  1_555      #   73 Check
PLAT743_ALERT_1_C Torsion Calc    -0.16(4), Rep       -0.02 ......    Missing s.u.
             N5  -C3  -N4  -C2    1_555  1_555  1_555  1_555      #   75 Check
PLAT743_ALERT_1_C Torsion Calc   110.83(4), Rep      110.76 ......    Missing s.u.
             N6  -C7  -O8  -O1    1_555  1_555  1_555  1_544      #   80 Check
```

Alert level G

```
PLAT004_ALERT_5_G Polymeric Structure Found with Maximum Dimension          1 Info
PLAT007_ALERT_5_G Number of Unrefined Donor-H Atoms .............          4 Report
             H1      H2      H3      H4
PLAT303_ALERT_2_G Full Occupancy Atom H4        with # Connections       2.00 Check
PLAT395_ALERT_2_G Deviating  X-O-Y   Angle From 120 for O8       .      154.6 Degree
PLAT710_ALERT_4_G Delete 1-2-3 or 2-3-4 Linear Torsion Angle ... #         2 Do !
             N1  -O1  -PD1 -O3   -179.52  0.00   1_555   1_555   1_555   1_555
PLAT710_ALERT_4_G Delete 1-2-3 or 2-3-4 Linear Torsion Angle ... #        12 Do !
             O1  -PD1 -O3  -C4     -0.40  0.00   1_555   1_555   1_555   1_555
PLAT710_ALERT_4_G Delete 1-2-3 or 2-3-4 Linear Torsion Angle ... #        16 Do !
             O2  -PD1 -O4  -C8     -0.24  0.00   1_555   1_555   1_555   1_555
PLAT710_ALERT_4_G Delete 1-2-3 or 2-3-4 Linear Torsion Angle ... #        50 Do !
             O4  -PD1 -O2  -N2   -179.84  0.00   1_555   1_555   1_555   1_555
PLAT710_ALERT_4_G Delete 1-2-3 or 2-3-4 Linear Torsion Angle ... #        51 Do !
             O4  -PD1 -O2  -H4    -68.98  0.00   1_555   1_555   1_555   1_566
PLAT710_ALERT_4_G Delete 1-2-3 or 2-3-4 Linear Torsion Angle ... #        67 Do !
             O8  -O1  -PD1 -O3     24.09  0.00   1_566   1_555   1_555   1_555
PLAT794_ALERT_5_G Tentative Bond Valency for Pd1       (II)      .       1.85 Info
PLAT804_ALERT_5_G Number of ARU-Code Packing Problem(s) in PLATON          1 Info
PLAT981_ALERT_1_G No non-zero f" Anomalous Scattering Values Found     Please Check
PLAT986_ALERT_1_G No non-zero f' Anomalous Scattering Values Found     Please Check
```

```
   5 ALERT level A = Most likely a serious problem - resolve or explain
   5 ALERT level B = A potentially serious problem, consider carefully
 102 ALERT level C = Check. Ensure it is not caused by an omission or oversight
  14 ALERT level G = General information/check it is not something unexpected

 103 ALERT type 1 CIF construction/syntax error, inconsistent or missing data
   9 ALERT type 2 Indicator that the structure model may be wrong or deficient
   3 ALERT type 3 Indicator that the structure quality may be low
   7 ALERT type 4 Improvement, methodology, query or suggestion
   4 ALERT type 5 Informative message, check
```

It is advisable to attempt to resolve as many as possible of the alerts in all categories. Often the minor alerts point to easily fixed oversights, errors and omissions in your CIF or refinement strategy, so attention to these fine details can be worthwhile. It is up to the individual to critically assess their own results and, if necessary, seek expert advice.

PLATON version of 15/01/2026; check.def file version of 02/01/2026

---

duplicate check

No duplication found

Datablock I - ellipsoid plot

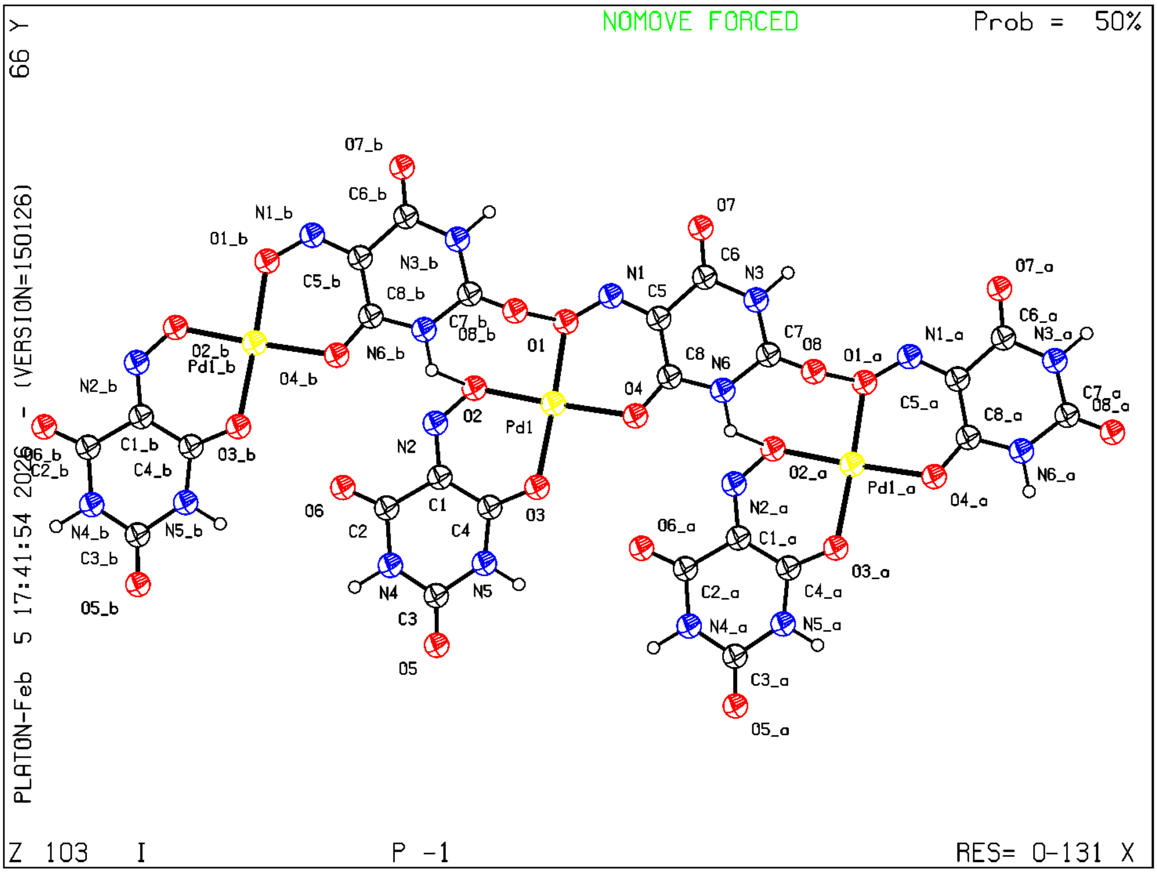

Supplement: Supplementary file 1 — Supplementary Material 1 [file 41598_2026_58248_MOESM1_ESM.pdf]
