# Supplementary material for: Synthesis, structural characterization, and dual DNA/HSA binding of novel Palladium(II) violurate complex with selective p53/Caspase-3-mediated anticancer activity
Source: Sci Rep. 2026 Jun 27;16:19632. doi: 10.1038/s41598-026-58248-w (PMC13310190; doi:10.1038/s41598-026-58248-w)
Supplement: Supplementary file 4 — Supplementary Material 4 [file 41598_2026_58248_MOESM4_ESM.doc]

Deposition Number

CCDC 2484420

(DOI:10.5517/ccdc.csd.cc2pd7ly)
