# Supplementary material for: Synthesis, structural characterization, and dual DNA/HSA binding of novel Palladium(II) violurate complex with selective p53/Caspase-3-mediated anticancer activity
Source: Sci Rep. 2026 Jun 27;16:19632. doi: 10.1038/s41598-026-58248-w (PMC13310190; doi:10.1038/s41598-026-58248-w)
Supplement: Supplementary file 5 — Supplementary Material 5 [file 41598_2026_58248_MOESM5_ESM.docx]

**Supplementary Materials**

**S1**: Physicochemical measurements, DNA/HSA-binding studies, viscosity measurements and method of DFT calculations

**S1.1.** **DNA – binding measurements**

UV–visible absorption spectroscopy was used to investigate the binding of violuric acid and its Pd(II) complex to ct-DNA. The stock solution of ct-DNA (6.25 ×10^-4^ M) was prepared by dissolving in Tris–HCl buffer solution (pH 7.2) and stored at a temperature of less than 4 °C. The stock solutions of violuric acid (6.25 × 10^-4^ M) and its Pd(II) complex (9 × 10^-4^ M) were prepared by using DMSO solvent. The binding experiments were carried out with the violuric acid at a concentration of (6.25 × 10^-5^ M) and its Pd(II) complex at a constant concentration of (9 × 10^-5^ M) while the ct-DNA quantities were varied (0.662252 × 10^-5^ to 4.4586 × 10^-5^ M).

Fluorescence quenching measurements were performed by using fixed amount of ct-DNA (6.25 ×10^-5^ M) and violuric acid with the varying concentrations from 6.21118 × 10^-6^ to 6.97674 × 10^-5^ M, while the Pd(II) complex concentration was varied between 9.28793 × 10^-6^ and 1.18457 × 10^-4^ M.

**S1.2.** **HSA – binding measurements**

Interactions of both free violuric acid and its Pd(II) complex with HSA have been studied by utilizing UV–visible absorption and fluorescence spectral techniques. The HSA solution was prepared by dissolving in Tris–HCl buffer solution (pH 7.2) and the violuric acid and Pd(II) complex were dissolved in DMSO solution. The absorption investigation was carried out by gradually increasing the quantity of HAS (6.0241×10^-6^ to 7.30337 × 10^-5^ M); keeping the fixed quantity (9 × 10^-5^ M) of violuric acid and Pd(II) complex. Furthermore, the fluorescence quenching experiment was performed by addition of gradually increasing of free violuric acid (from 1.2945×10^-5^ to 1.15942×10^-4^ M) and Pd(II) complex of concentration from 9.74026 × 10^-6^ to 1.10787 × 10^-4^ M into a fixed concentration (1.64 ×10^-5^ M) of the HSA solution.

**S1.3. Viscosity titration measurements**

Viscosity experiments were conducted on an Ubbelodhe viscometer, immersed in a water bath maintained at 25.0 ± 0.1 ºC. The flow time was measured with a digital stopwatch and each sample was tested, three times to get an average calculated time. Titrations were performed for the free violuric acid and its Pd(II) complex (3 – 30 μM), and each compound was introduced into ct-DNA solution (42.5 μM) present in the viscometer. Data were analyzed as (η/η_0_)^1/3^ versus the ratio of the concentration of the compound to ct-DNA, where η is the viscosity of ct-DNA in the presence of the compound and η_0_ is the viscosity of ct-DNA alone. Viscosity values were calculated from the observed flow time of ct-DNA-containing solutions corrected from the flow time of buffer alone (t_0_), η = (t – t_0_) [1].

**S1.4. Geometry optimization with the DFT technique**

All calculations were performed using density functional theory (DFT) as implemented in Gaussian 09. [2] The hybrid B3LYP approach [3–5] was used for geometry optimization. Mixed basis sets were used, for which Cu atom was treated with LanL2DZ basis sets, while all-electron 6-31G(D) basis sets were used for other elements in the complex.

**S1.5. Rietveld refinement**

Initially, the starting structure of the synthesized Pd(II) complex was obtained from the optimized structure generated by DFT calculations using Gaussian 09 software. The first step in solving the structure from powder diffraction data is indexing, which determines crystallographic information like unit cell parameters. EXPO 2014 performs the indexing using the N-TREOR09 program (Altomare et al., 2009), followed by space group determination through careful analysis of the experimental intensities.

The crystal structure was then solved by powder diffraction through the simulated annealing (SA) method, which involves generating random trial structures based on the optimized 3D model and adjusting them until the calculated pattern matches the observed data. The best-fit structure was refined using the Rietveld method, refining parameters such as peak shape, unit cell dimensions, and scale factor, while maintaining complete restraints on bond lengths, angles, and planes. A Pseudo-Voigt function was used to model the peak shape, with a maximum of 30 refinement cycles conducted. The non-linear least squares refinement was performed using the damped Gauss-Newton algorithm.


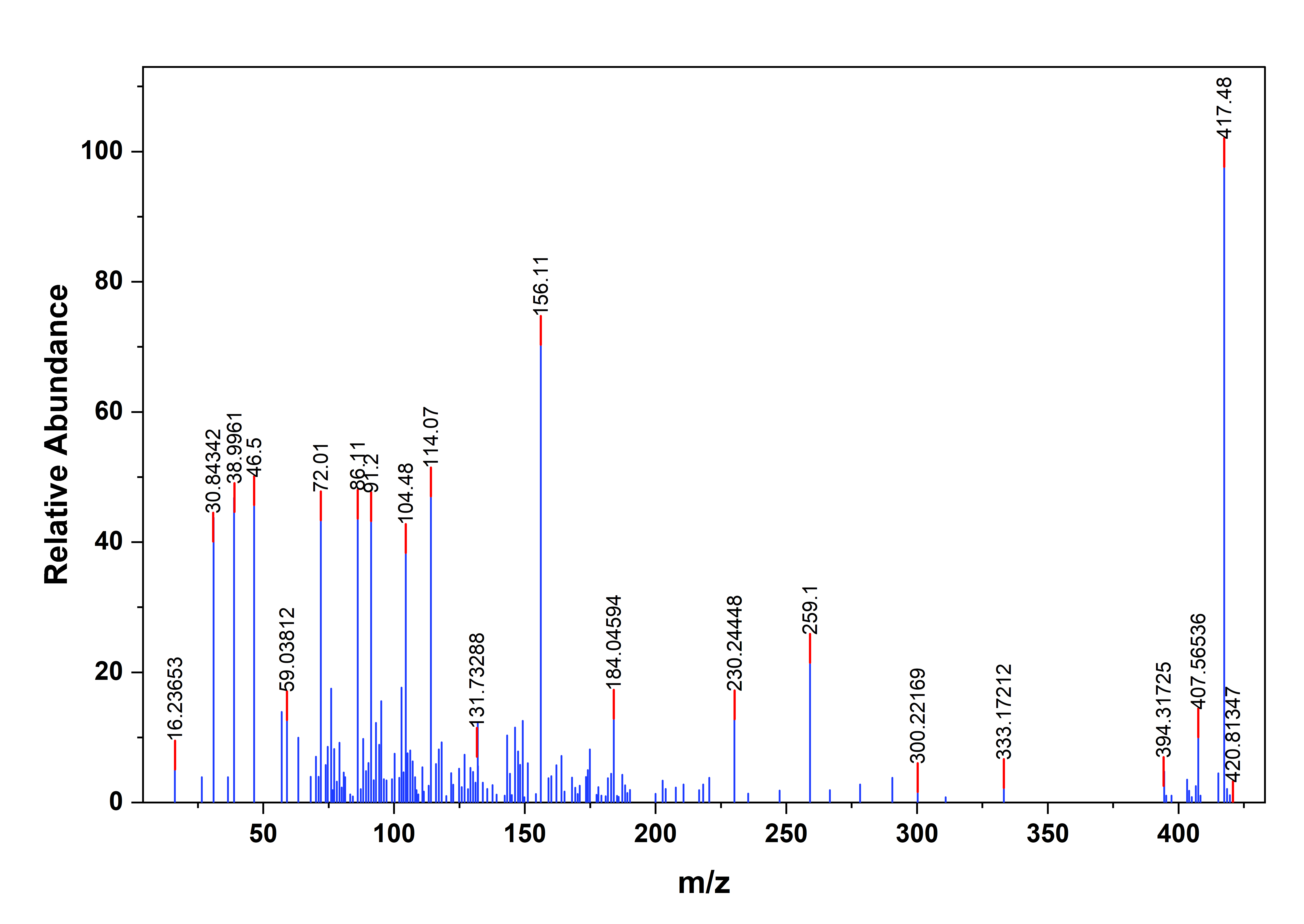


Fig S2: EI-MS spectrum of violurate – based Pd(II) complex, [Pd(H_2_L)_2_]


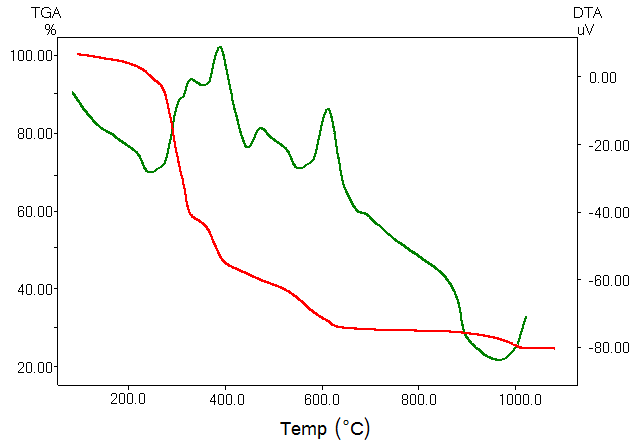


Fig S3: Thermogravimetric (TGA) and differential thermal analysis (DTA) diagram of [Pd(H_2_L)_2_] complex


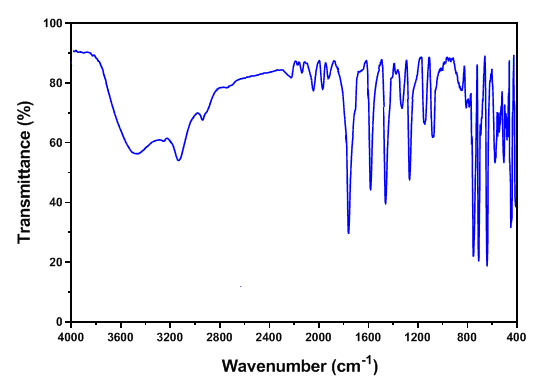


Fig S4: FTIR spectrum of the violuric acid


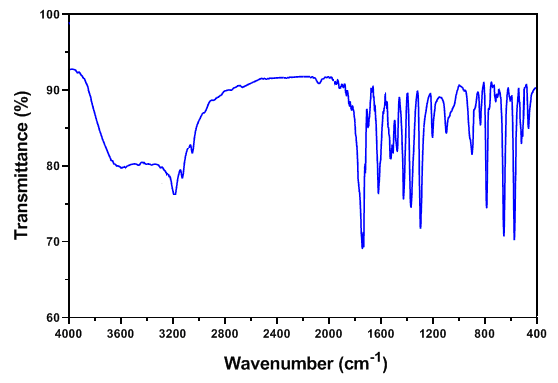


Fig S5: FTIR spectrum of the [Pd(H_2_L)_2_] complex


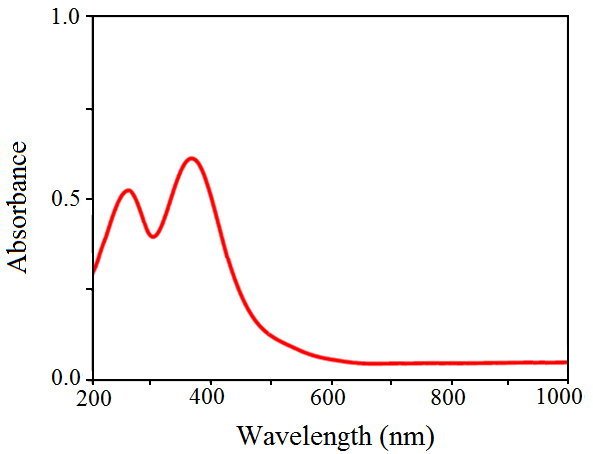


Fig S6: Electronic absorption spectrum of free violuric acid


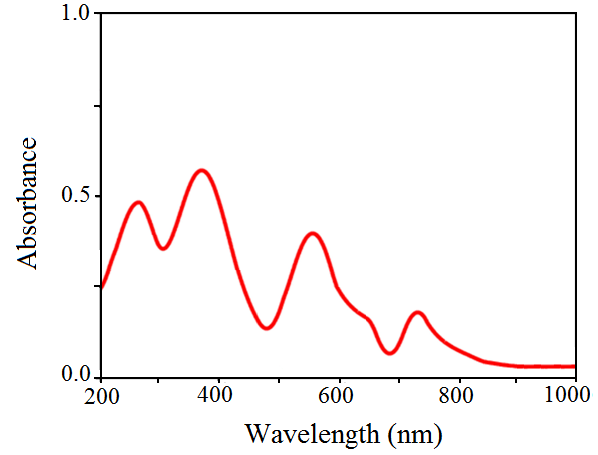


Fig S7: Electronic absorption spectrum of [Pd(H_2_L)_2_] complex

Table S8: Electronic absorption spectral features (nm) of violuric acid its Pd(II) complex [Pd(H_2_L)_2_]

| Compound | ^1^*A*_1g_ → ^1^*A*_2g_ (ν_1_) | ^1^*A*_1g_ → ^1^*B*_1g_ (ν_2_) | ν_1_/ ν_2_ | π → π* | *n* → π* |
| --- | --- | --- | --- | --- | --- |
| Free H_3_L* | - | - | - | 262 | 340 |
| [Pd(H_2_L)_2_] | 560 | 730 | 0.77 | 270 | 355 |

* Violuric acid


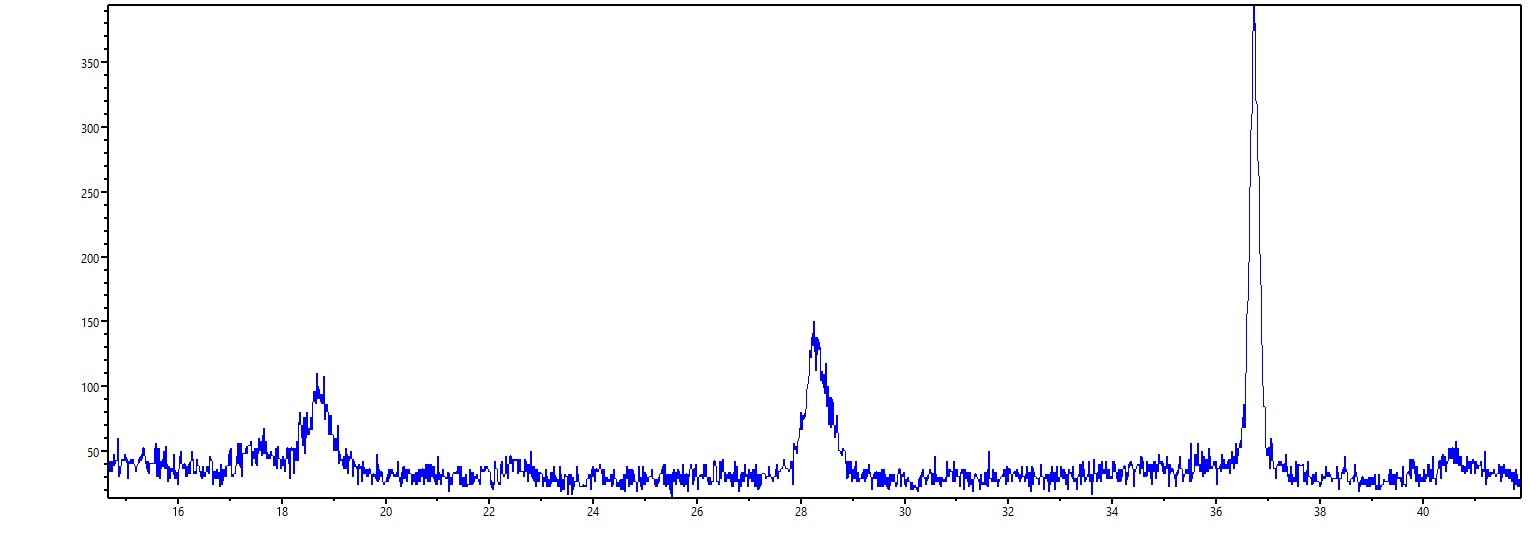


Fig S9: PXRD spectrum of bis-chelate palladium(II) violurate complex, [Pd(H₂L)₂]


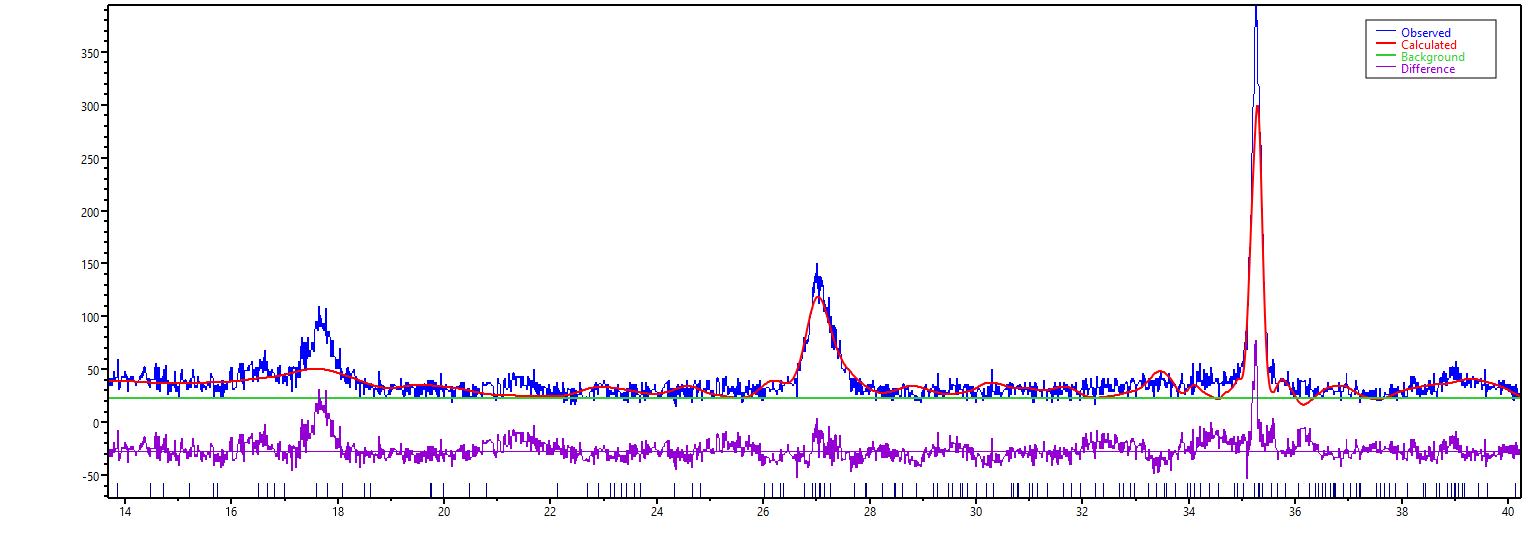


Fig S10: The maximize fit between the XRD spectroscopic data and the computationally generated data

Table S11: Global reactivity descriptors of violuric acid and its Pd(II) complex, [Pd(H_2_L)_2_]

| **Property** | **[Pt(H_2_L)_2_]** | **(H_3_L)** |
| --- | --- | --- |
| **E_LUMO_** | -1.99732 | -1.3382 |
| **E_HOMO_** | -7.40286 | -7.855528 |
| **ΔE (eV)** | 5.405546 | 6.51723729 |
| **χ** | 4.70009 | 4.596910262 |
| **η** | 2.702773 | 3.258618645 |
| **S** | 0.184995 | 0.15343925 |
| **P_i_** | -4.70009 | -4.596910262 |
| **ω** | 4.086701 | 3.242414388 |
| **ΔN_max_** | 1.738988 | 1.410692923 |
| **μ** | -5.3439 | -4.5969 |


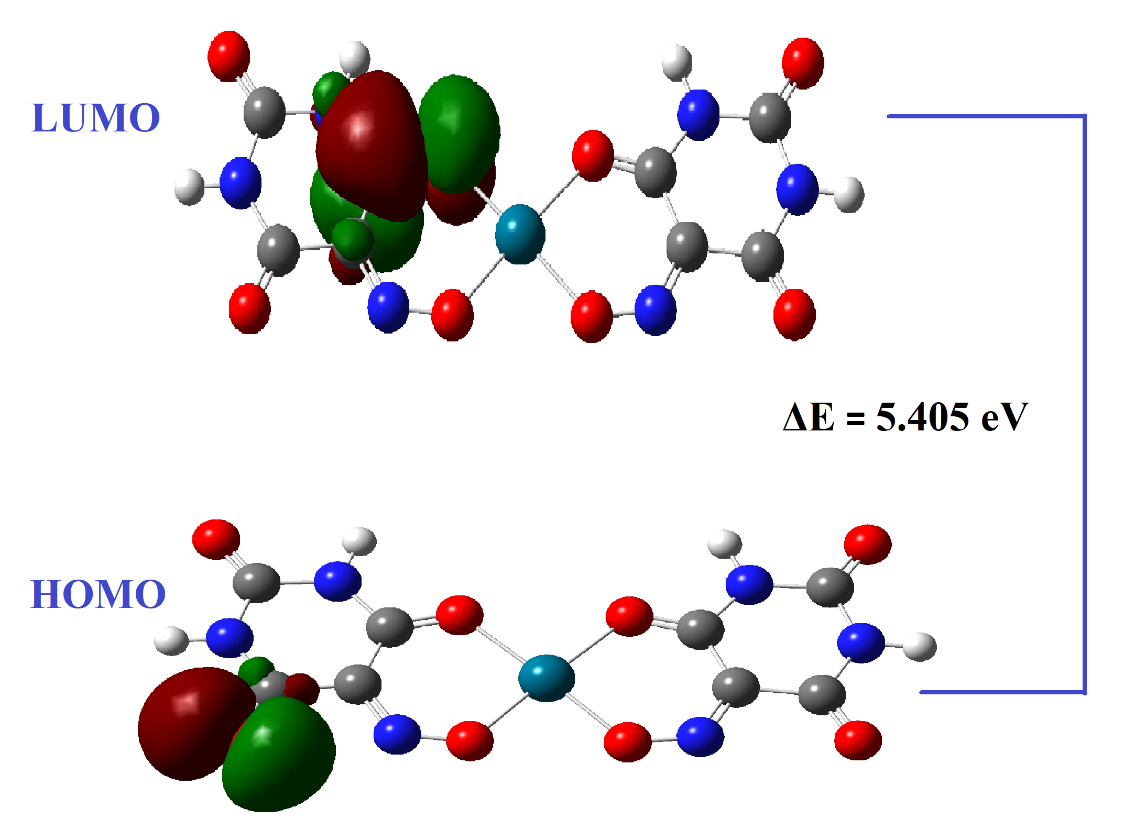


Fig S12: Frontier Molecular Orbitals (FMOs) for Pd(II) complex


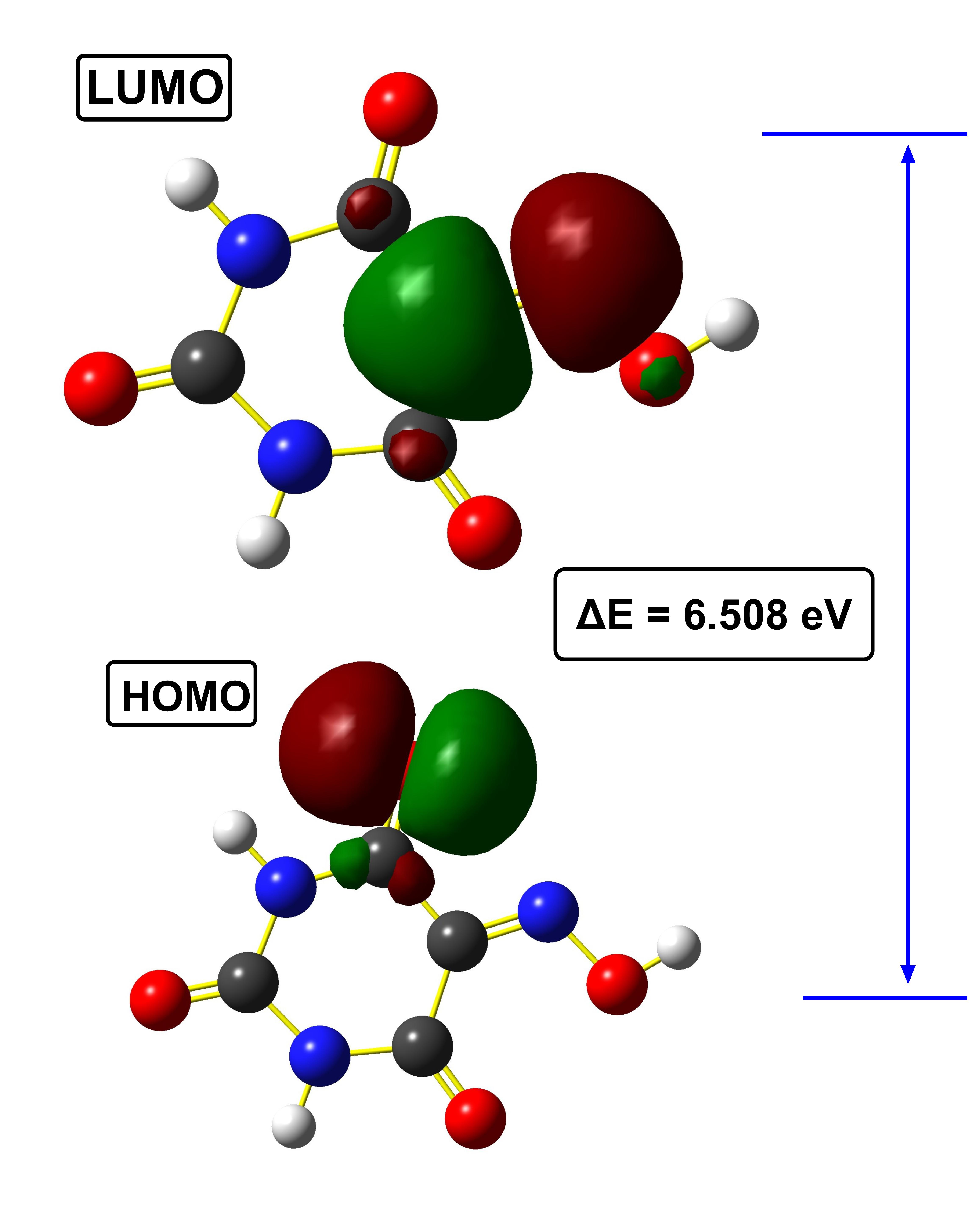


Fig S13: Frontier Molecular Orbitals (FMOs) for Violuric acid

Fig S14: UV-spectral profile of violuric acid binding with DNA

Fig. S15: Fluorescence quenching spectral profile of violuric acid binding with DNA


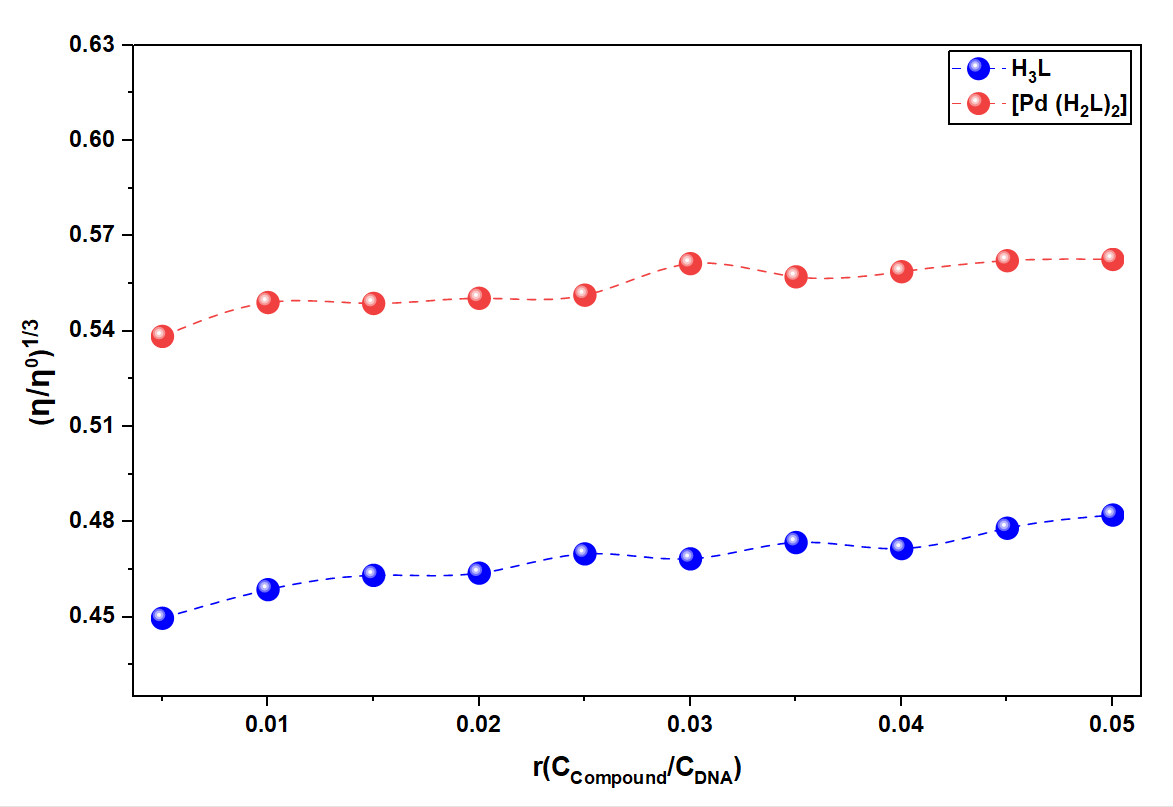


Fig. S16: Effects of incremental additions of free violuric acid (H₃L) and its Pd(II) complex, [Pd(H₂L)₂], on the relative viscosity of DNA solutions

Fig. S17: UV-spectral profile of violuric acid binding with HSA

Fig. S18: Fluorescence quenching spectral profile of violuric acid binding with HSA


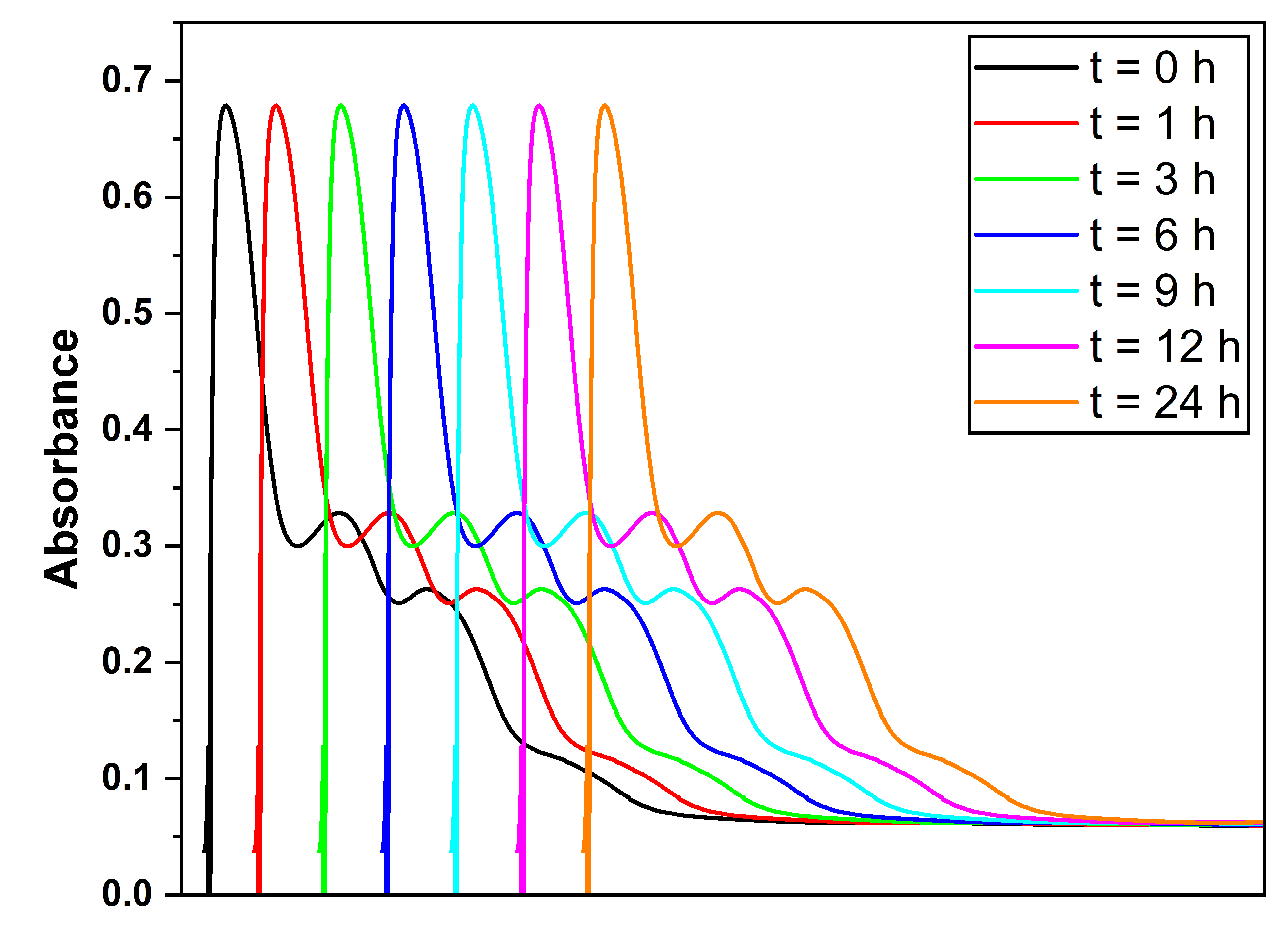


Fig S19: The electronic absorption spectral profile of [Pd(H_2_L)_2_] complex in tris-HCl buffer solution at 270 and 355 nm over time periods ranging from zero minutes to 24 hours

| 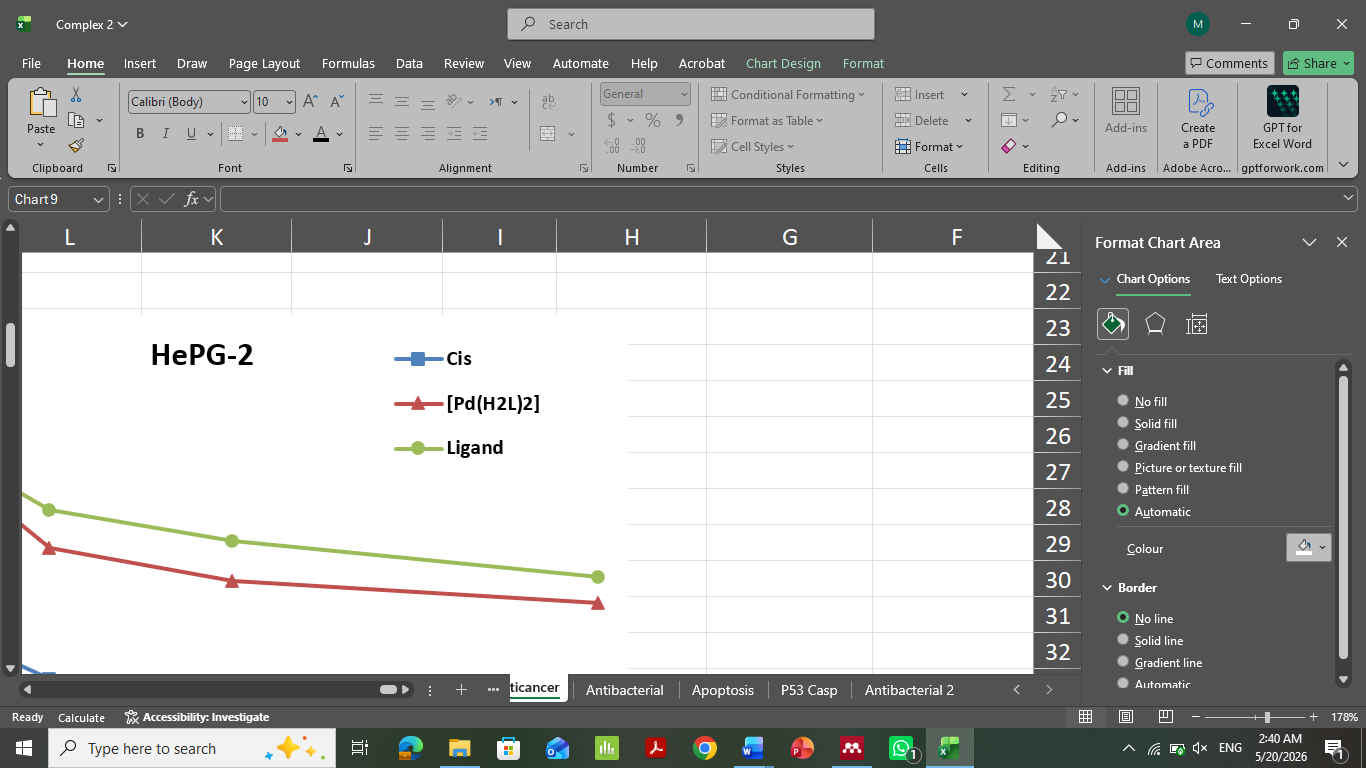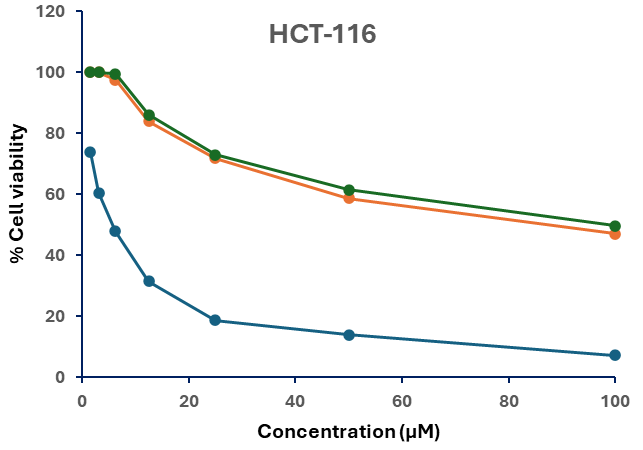 | 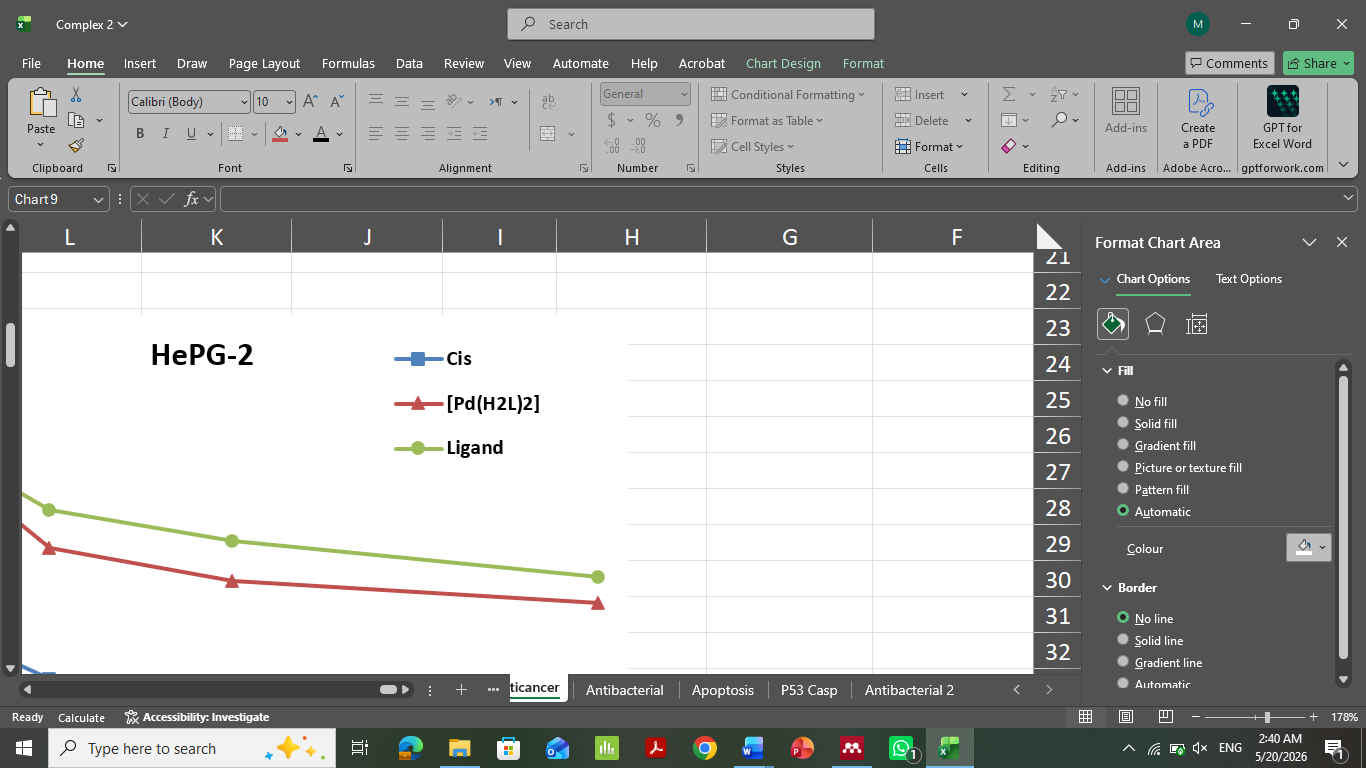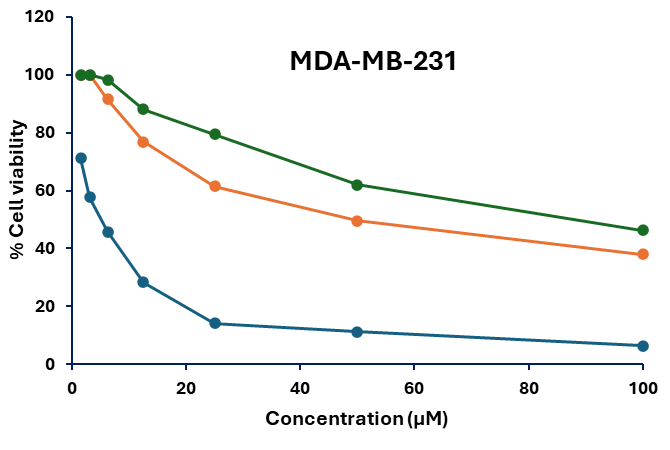 |
| --- | --- |

Fig. S20: Viability testing of HCT-116 cells (B) and MDA-MB-231 cells (C) against different concentrations of [Pd(H_2_L)_2_], ligand, and Cis.

Table S21. Selectivity indices (SIs) for free VA, [Pd(H_2_L)_2_], and cisplatin

| cisplatin | [Pd(H_2_L)_2_] | Free VA | SI |
| --- | --- | --- | --- |
| 0.742 | 1.54 | 0.529 | IC_50_ of WISH cell/IC_50_ of HepG-2 cell |
| 0.638 | 0.896 | 0.453 | IC_50_ of WISH cell/ IC_50_ of HCT-116 cell |
| 1.05 | 1.96 | 0.505 | IC_50_ of WISH cell/ IC_50_ of MDA-231 cell |

**■ Viable ■ Early apoptosis**

**■ Late apoptosis ■ Necrosis**

*

*

*^#^

*^#^


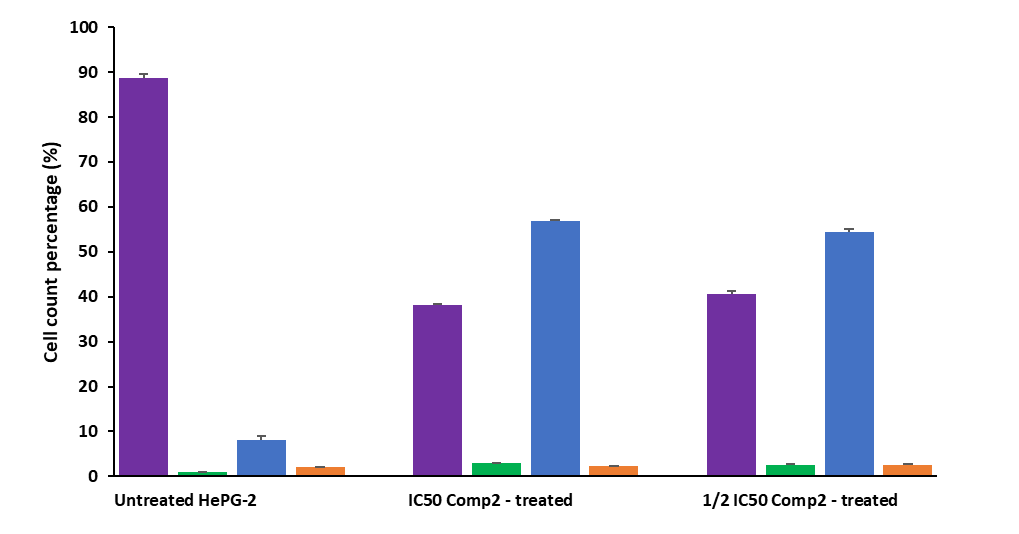


Figure S22: Bar plot of the percentages of early and late apoptotic cell death in untreated HePG-2 cells versus those treated with the IC_50_ and ½ IC_50_ of Comp 2. * *p <* 0.05 significant compared to untreated HePG-2 cells, and ^#^ *p <* 0.05 significant compared to HePG-2 cells treated with ½ IC_50_ of [Pd(H_2_L)_2_].

| **1 2 3** 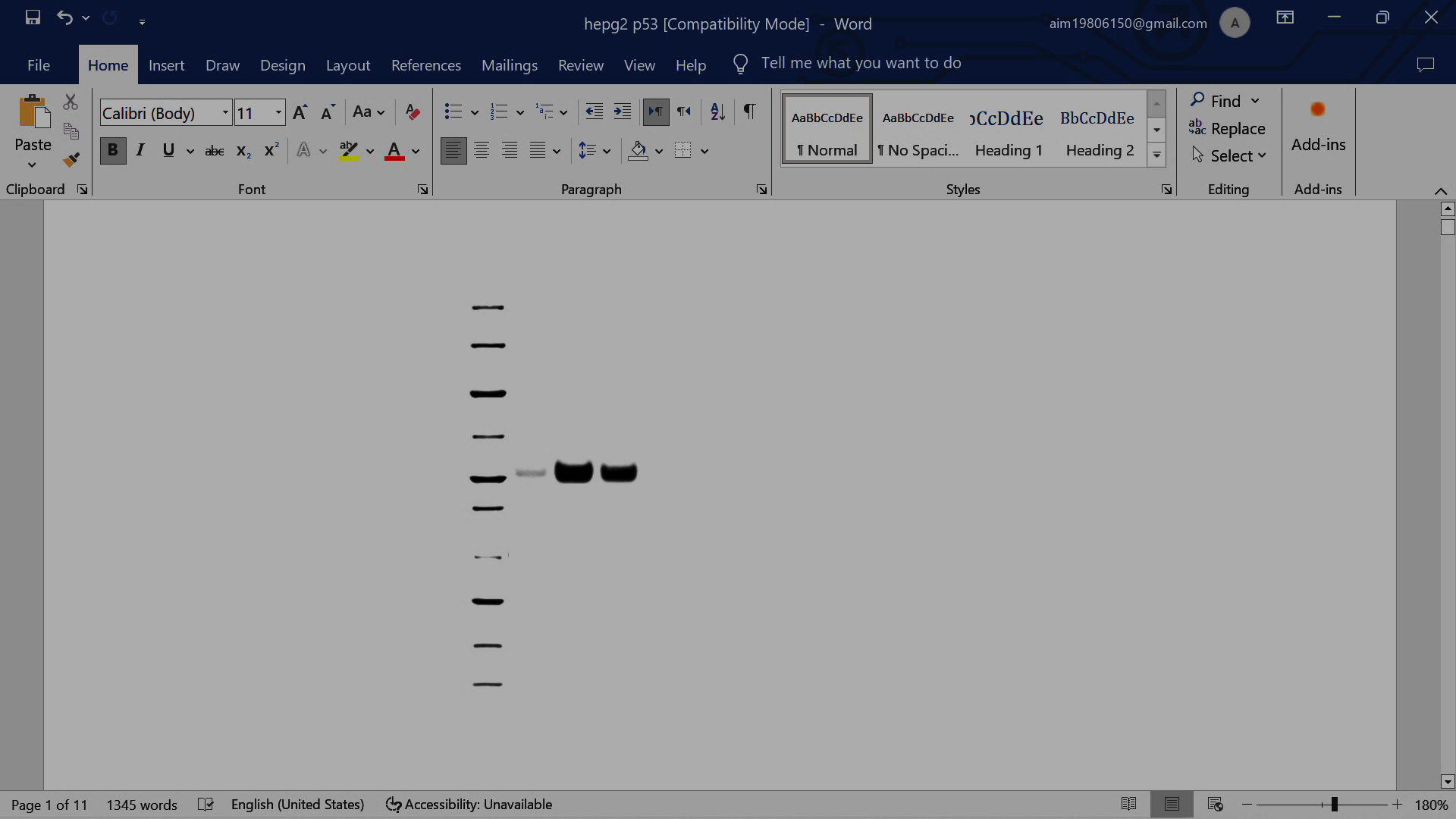 | 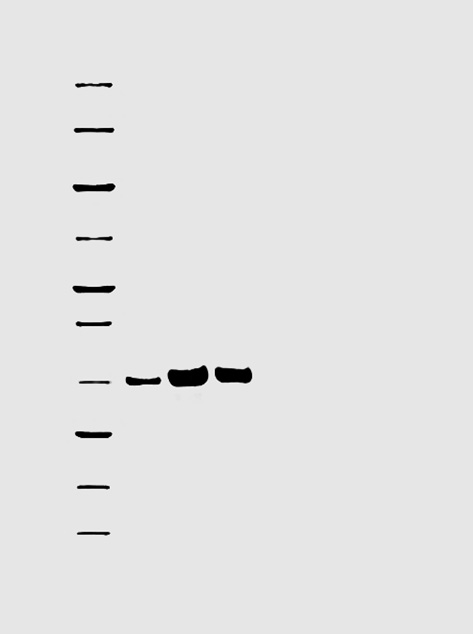 |
| --- | --- |
| P53 expression with the marker protein (first lane), HePG-2 untreated cells (2^nd^ lane), and [Pd(H_2_L)_2_] treated cells (3^rd^ lane). | Caspase 3 with the marker protein (first lane), HePG-2 untreated cells (2^nd^ lane), and [Pd(H_2_L)_2_] treated cells (3^rd^ lane). |
| **KDa**  **250**  **150**  **100**  **70**  **50**  **40**  **30**  **20**  **15**  **5** 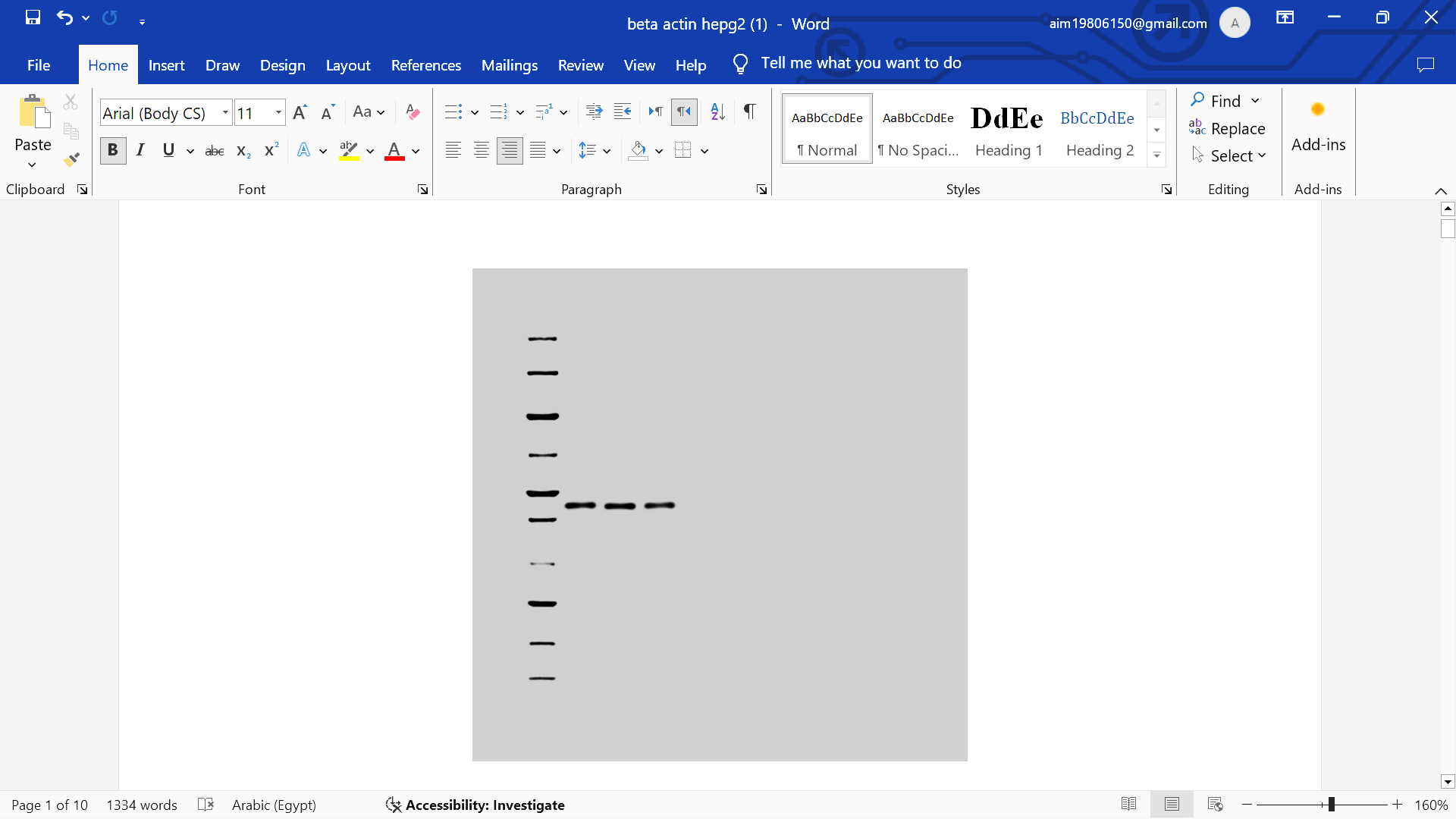 **1 2 3** | |
| β-actin (housekeeping protein) expression with the marker protein (first lane), HePG-2 untreated cells (2^nd^ lane), and [Pd(H_2_L)_2_] treated cells (3^rd^ lane). | |

Fig. S23: Western blot original gel image. In every membrane, the red rectangle represents and shaped the bands used in our study.

**1 2 3**

**KDa**

**250**

**150**

**100**

**70**

**50**

**40**

**30**

**20**

**15**

**5**

**KDa**

**250**

**150**

**100**

**70**

**50**

**40**

**30**

**20**

**15**

**5**

**References**

1. Gaur, R. *et al.* Interaction of a ruthenium(II) chalcone complex with double stranded DNA: Spectroscopic, molecular docking and nuclease properties. *J. Photochem. Photobiol. A Chem.* **220**, 145–152 (2011).

2. Frisch, M. J. *et al.* Gaussian09 Revision A.02. *Gaussian, Inc., Wallingford CT* at (2009).

3. Becke, A. D. Density-functional thermochemistry. III. The role of exact exchange. *J. Chem. Phys.* **98**, 5648–5652 (1993).

4. Lee, C., Yang, W. & Parr, R. G. Development of the Colle-Salvetti correlation-energy formula into a functional of the electron density. *Phys. Rev. B* **37**, 785–789 (1988).

5. Stephens, P. J., Devlin, F. J., Chabalowski, C. F. & Frisch, M. J. Ab Initio Calculation of Vibrational Absorption and Circular Dichroism Spectra Using Density Functional Force Fields. *J. Phys. Chem.* **98**, 11623–11627 (1994).
